# Global trends in tumor-associated neutrophil research: a bibliometric and visual analysis

**DOI:** 10.3389/fimmu.2025.1478092

**Published:** 2025-03-14

**Authors:** Shaodong Li, Peng Dong, Xueliang Wu, Zhenhua Kang, Guoqiang Yan

**Affiliations:** ^1^ Department of Hepatobiliary and Pancreatic Surgery, General Surgery Center, The First Hospital of Jilin University, Changchun, China; ^2^ Department of Anesthesiology, Nanfang Hospital, Southern Medical University, Guangzhou, China; ^3^ Department of General Surgery, The First Affiliated Hospital of Hebei North University, Zhangjiakou, China; ^4^ Department of Colorectal & Anal Surgery, General Surgery Center, First Hospital of Jilin University, Changchun, China

**Keywords:** tumor-associated neutrophil, tumor microenvironment, VOSviewer, CiteSpace, visual analysis

## Abstract

**Background:**

Tumor-associated neutrophils (TANs) play crucial roles in tumor progression, immune response modulation, and the therapeutic outcomes. Despite significant advancements in TAN research, a comprehensive bibliometric analysis that objectively presents the current status and trends in this field is lacking. This study aims to fill this gap by visually analyzing global trends in TANs research using bibliometric and knowledge mapping techniques.

**Methods:**

We retrieved articles and reviews related to TANs from the Web of Science core collection database, spanning the period from 2012 to2024. The data was analyzed using bibliometric tools such as Excel 365, CiteSpace, VOSviewer, and Bibliometrix (R-Tool of R-Studio) to identify key trends, influential countries and institutions, collaborative networks. and citation patterns.

**Results:**

A total of 6l5 publications were included in the bibliometric analysis, showing a significant upward trend in TANs research over the last two decades. The United States and China emerged as the leading contributors with the highest number of publications and citations. The journal with the most publications in this field is Frontiers in Immunology, Prominent authors such as Fridlender ZG was identified as the key contributor, with his works frequently cited. The analysis highlighted major research themes. including the role of TANs in tumor microenvironment modulation, their dual functions in tumor promotion and suppression, and the exploration of TANs-targeted therapies, Emerging research hotspots include studies on TANs plasticity and their interactions with other immune cells.

**Conclusion:**

This study is the first to employ bibliometric methods to visualize trends and frontiers in TANs research. The findings provide valuable insights into the evolution of the field, highlighting critical areas for future investigation and potential collaborative opportunities. This comprehensive analysis serves as a crucial resource for researchers and practitioners aiming to advance TAN research and its application in cancer therapy.

## 1 Introduction

Neutrophils, originating from Granulocyte-Monocyte Progenitors (GMPs) cells in the bone marrow (1, 2), play a key role in the innate immune system of the human body, which is not only involved in defending against infections, regulating inflammatory responses and tissue repair (3–5), but also in tumorigenesis and progression that has been progressively recognized by the scientific community in recent years. In the complex and dynamically changing environment of the tumor microenvironment (TME), neutrophils are recruited and transformed into tumor-associated neutrophils (TANs) with varying degrees of maturity and tissue affinity in response to a variety of chemokines secreted by tumor cells (6, 7), such as C-X-C motif chemokine [e. g., interleukin-8 (IL-8)], which exhibit significant heterogenous (8).

TANs have a diversity and plasticity that enables them to exhibit anti-tumor or pro-tumor properties depending on the signals in the TME and are accordingly classified into anti-tumor N1 TANs and protumorigenic N2 TANs (9). However, no specific surface markers have been identified to differentiate between these two subtypes, and thus TANs are mainly defined by their functional phenotype (10). TANs mentioned above exert their anticancer effects through different mechanisms, including through antibody dependent cell-mediated cytotoxicity (ADCC), direct cytotoxicity through the release of cytotoxic mediators such as ROS, myeloperoxidase (MPO), and the activation of adaptive anti-tumor immune response (11–13); some studies have shown that, in comparison to N2 TANs, anti-tumor N1 TANs have been shown to produce higher levels of Tumor necrosis factor i *α* (TNF-*α*), MIP-1a, H2O2, and NO, and to be cytotoxic to tumor cells *in vitro* and *in vivo* (14, 15); TANs and neutrophil extracellular traps (NETs) also interact to promote immune evasion in a PD-L1/PD-1-dependent interaction, a phenomenon widely recognized in pancreatic cancer (16, 17).

The formation of TANs is a finely regulated process involving multiple cytokines, signaling pathways, and microenvironmental factors. In terms of phenotypic polarization, the formation of N1-type TANs may be associated with the exposure of cytokines such as interferon (IFN) and TNF-*α* (14, 18), which promote anti-tumor immune response. In contrast, the formation of N2-type TANs is associated with factors such as transforming growth factor-*β* (TGF-*β*) (19), which tend to promote angiogenesis and immune escape, thereby favoring tumor progression. In TME, neutrophils may also be affected by other cytokines, direct contact with tumor cells, and other immune cell interactions, which together result in a shift in neutrophil phenotype (20–22). Specific signaling pathways, such as phosphatidylinositol 3-kinase (PI3K)/Akt, MAPK, and NF-*κ*B (23), may play a key role in this process. Meanwhile, microenvironmental factors such as hypoxia, acidosis and nutrient supply may also affect neutrophil phenotype and function by influencing neutrophil metabolism and signaling (24).

The double-edged nature of TANs has important implications for tumor progression and patient prognosis. Although early studies of TANs focused on anticancer effects, a growing body of research suggests that TANs often exhibit a pattern in TME that is more similar to the N2-type tumor-promoting phenotype. The fact that tumor cells themselves mediate neutrophil recruitment to the site of tumorigenesis by secreting CXC chemokines also strongly suggests that TANs are not an effective means of host antitumor activity. Both neutrophil depletion assays and tumor site neutrophil accumulation inhibition assays have been shown to prevent tumor trophic vasculature (25–27) and inhibit tumor growth. Meanwhile, some clinical studies have shown that the presence of neutrophils leads to a poor prognosis. For example, elevated levels of PMNs in the fine bronchioloalveolar space of patients with bronchioloalveolar carcinoma were significantly associated with poor prognosis (28). These findings illustrate that although TANs have the potential to fight tumors by activating the immune system, they are more likely to be manipulated by tumor cells to promote tumor growth and spread. Their role as potential therapeutic targets for tumors requires a deeper exploration of the dual role of TANs in tumor immunity. How to reduce the production of N2-type as well as increase the expression of N1-type may be a hot research direction in the future.

The plasticity and heterogenous of TANs allow TANs to promote or inhibit tumor growth and progression through a variety of complex pathways; therefore, quantitative evaluation and analysis of the current status of research, focus areas, and development trends of TANs is essential for understanding their role in tumor development. Bibliometrics is a cross-cutting science that quantitatively analyses all knowledge carriers using mathematics and statistics (29). It combines mathematics, statistics, and bibliography into a comprehensive body of knowledge that focuses on quantification and allows for the evaluation of systematic criteria in the field of medical research (30). Bibliometrics can provide researchers with a comprehensive and objective perspective. Not only can it identify the history and future trends of a specific field, but it can also systematically assess the research progress of different countries, institutions, and researchers (31, 32). The aim of this study was to explore the past research on tumor-associated neutrophils through bibliometrics and to provide new perspectives and directions for future research on TANs and to find the next research hotspot of TANs.

## 2 Methods

### 2.1 Search strategies

Data were extracted from the Web of Science Core Collection (WOSCC) database, one of the most widely used sources for academic and bibliometric analyses (33). The search formula was TS= (“tumor associated Neutrophil*”) OR (“tumor-associated Neutrophil*”) OR (“tumor associated Neutrophil*”) OR (“tumor-associated Neutrophil*”) OR (“ cancer associated Neutrophil*”) OR (“cancer-associated Neutrophil*”), set the publication period of the article to be from 2000 to 2024, limited the language to English. The publication type was limited to article and review article, and a total of 615 articles were retrieved. All retrieved documents were saved as records, and citations were output in the form of all records and citations, saved as plain text files and stored, and the study was completed on March 21, 2024, in order to ensure the accuracy of the data and to prevent data bias due to database updates. We completed the retrieval and collection of all data on 3/21/2024.

### 2.2 Data collection

Raw data were extracted from selected publications, including Abstract, Author(s), Title, Source, Times Cited Count, Accession Number, Authors Identifiers, ISSN/ISBN, PubMed ID, Conf. Info/Sponsors, Addresses, Affiliations, Document Type, Keywords, WOS Categories, Research Areas, WOS Editions (print only), Cited References, Cited Reference Count, Hot Paper, Highly Cited, Usage Count, Funding Information, Publisher Information, Open Access, Page Count, Source Abbrev. Number, Language, Publication year, References, Keywords, Researcher’s h-index, Journal Impact Factor (IF), and Journal Citation Report (JCR) divisions were obtained from Web of Science. The productivity of the paper was measured by the number of citations. Duplicate articles were merged into one element and misspelled words were corrected artificially. Cleaned data was exported for further analysis.

### 2.3 Bibliometric analysis and visualization

Bibliometric analysis and visualization are important tools for revealing trends and knowledge structures in research areas. By using tools such as R software, VOSviewer, and CiteSpace, we can analyze large amounts of bibliometric data in depth to derive valuable insights. We used R software to perform Lotka’s Law analysis to explore the distribution of authors’ publication frequencies (34). Lotka’s Law reveals the relationship between a small number of highly prolific authors and a large number of less prolific authors in scientific fields (35). The statistical analysis function of the R software allows us to test the applicability of this law in the study of TANs and to identify high-productivity authors and key literature, which is important for understanding the knowledge production and dissemination patterns of TANs. By using the VOSviewer tool, we constructed a scientometric network graph that presents the elements of the literature data such as keywords, authors, institutions or journals as nodes in the network graph (36). The size of a node is determined by its frequency of occurrence in the literature or the number of publications, while the connections between nodes reflect their relevance (37). By looking at the clustering of the nodes, we can identify closely related research topics or areas, while the thickness of the connections shows the strength of collaboration or citation between these nodes. In addition, Total link strength (TLS) represents the number of co-occurrences, which to some extent can reflect the collaborative exchange relationship between countries, organizations, and authors. By calculating and comparing the Total link strength, we can identify the key nodes that have an important position in the research field of TANs, and these key nodes may be the leaders, core institutions, or influential journals in the research field. Finally, with CiteSpace software, we can visualize and analyze the literature citation relationships in the research field (38). CiteSpace can help us track the evolution of research hotspots and identify key nodes and outbreaks in the research field. By analyzing the citation network and co-occurring keywords, we can reveal the development trend of TANs-related research and potential research opportunities.

## 3 Results

**Figure 1:**
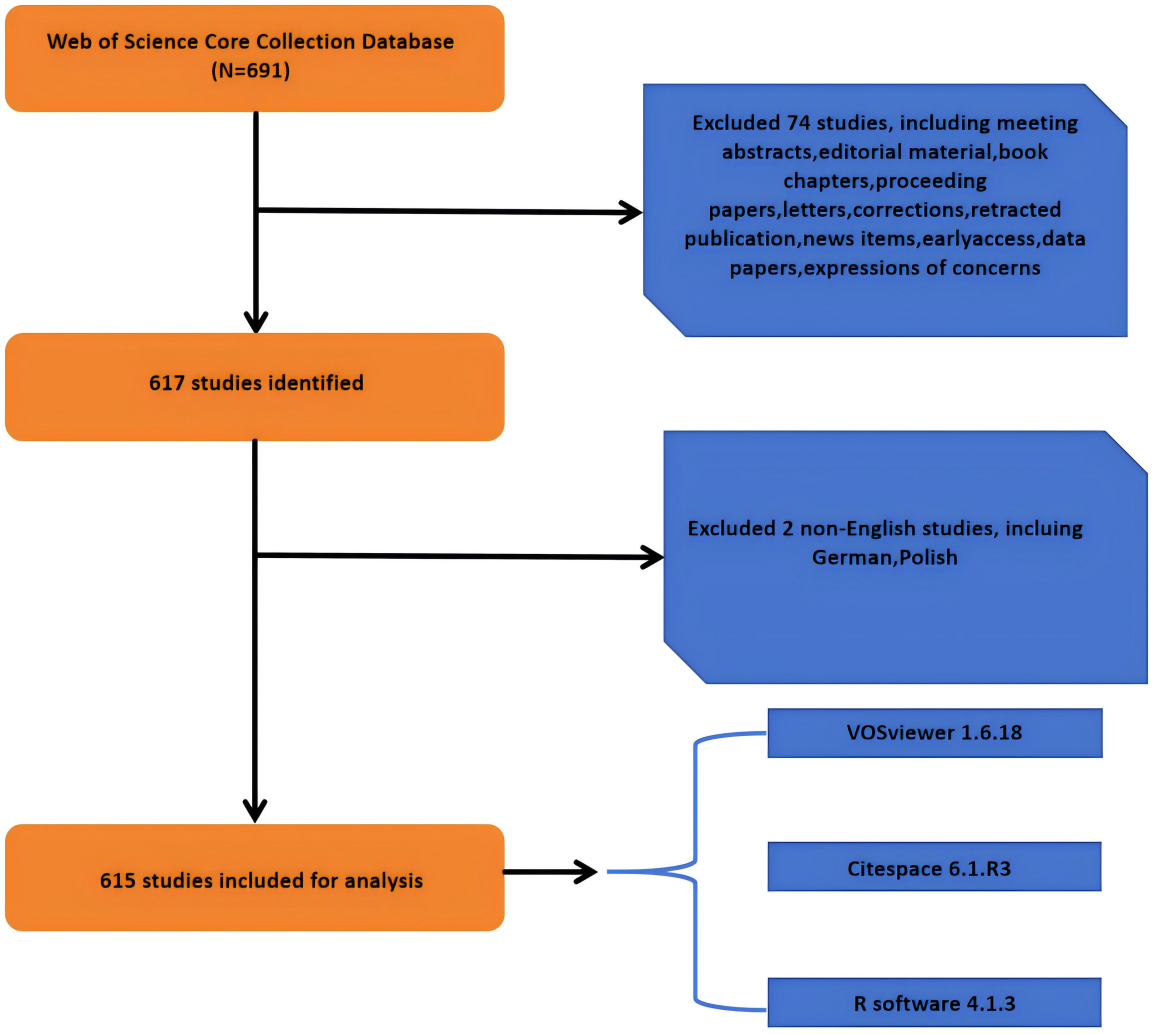
Flowchart of literature screening.

Between 2000 and 2024, a total of 691 articles were published in the field. Based on the exclusion criteria, we finally included 615 eligible original articles in our study. The specific flow chart is shown in **Figure 1**.

### 3.1 Trends in literature publishing output

**Figure 2:**
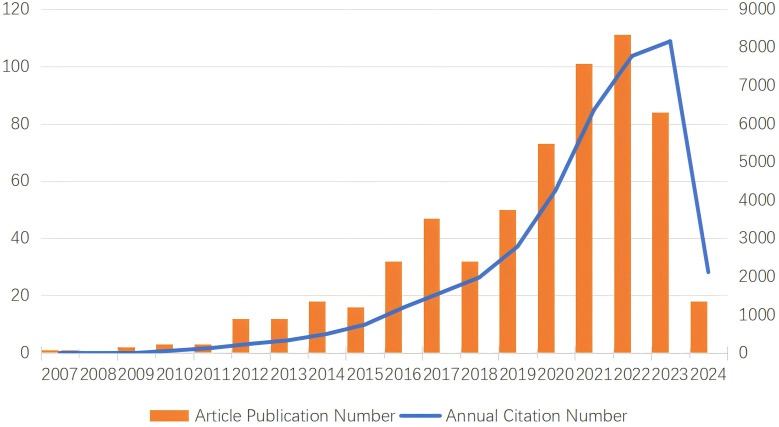
Description of TANs-related publication volume and citation frequency and publication volume.

The number of papers published in each period reflects the research trends in the field. As shown in **Figure 2**, for research on TAN, from 2007 to 2024, the overall trend has shown a steady increase, although the number of articles published has fluctuated in some periods. From 2009, when the N1/N2 functional classification of TANs was formally proposed by Fridlender ZG et al. (39), to 2015, the output of TANs-related literature was extremely low, with fewer than 20 articles per year, suggesting that research remained stagnant (**Figure 2**). From 2015 to 2024, the number of publications increased exponentially, with 548 articles published on TANs, representing 89.1% of the last two decades. This represents a surge in TANs research, indicating that TANs research has entered a period of rapid development in recent years. This may be related to the fact that TME-related studies have become hotspots and neutrophils have gradually gained importance in tumors. We collected 615 relevant studies in the field of TANs research between January 2000 and 2024 from the Web of Science database. Among them, the global citation score (GCS) was 37,374, and the average citation score per item was 60.77, and the global citation score was as high as 8,716 in 2022, which may be a breakthrough in this field of research. After 2015, this research was in a rapid development stage, and the number of annual publications gradually increased.

### 3.2 Distribution of countries/regions

**Figure 3:**
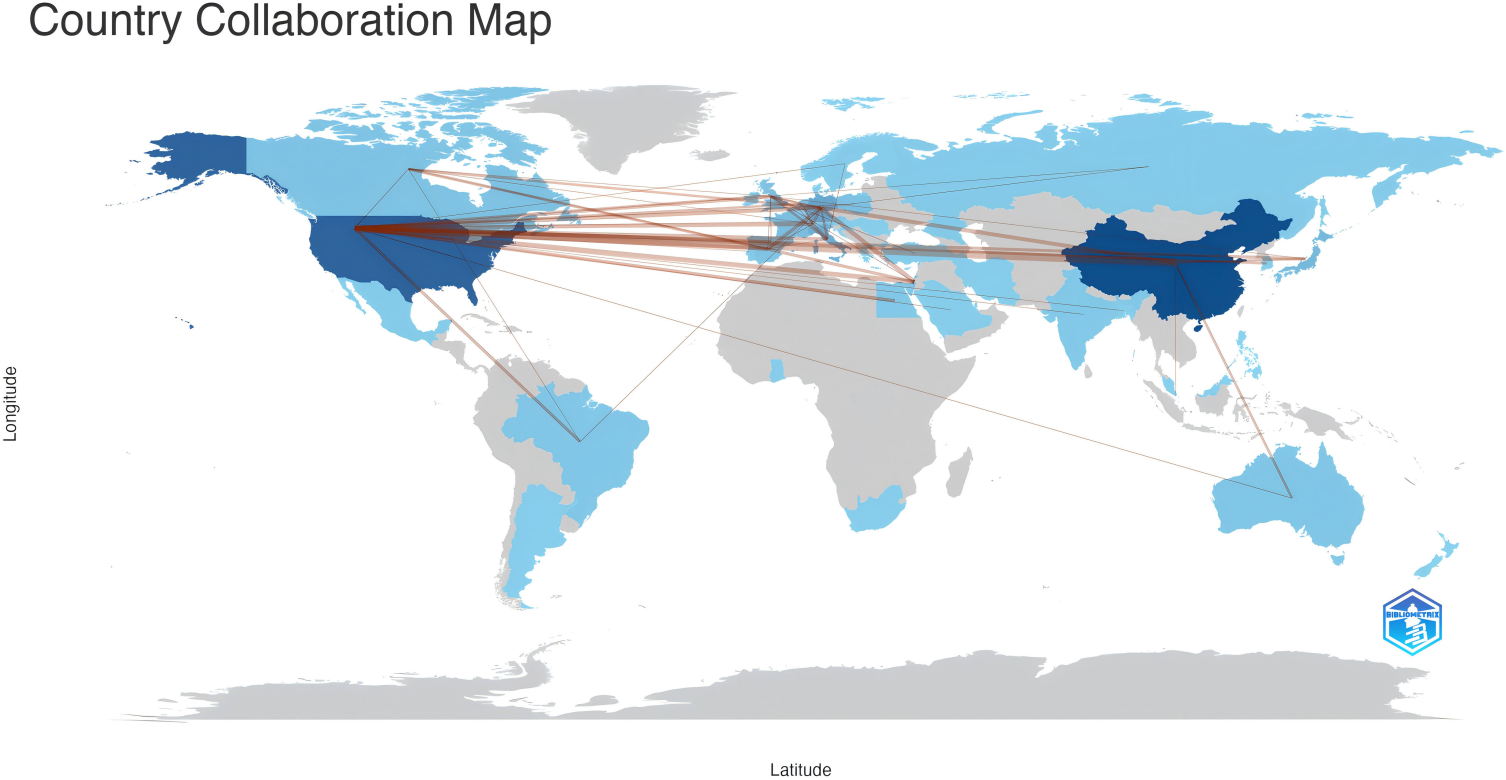
Countries/regions involved in TANs-related research. The links between countries/regions indicate their collaborations and connections, where the collaboration count between any two countries must be greater than 2.

From 51 countries/regions involved in TANs, **Table 1** shows the 10 countries/regions with the highest number of publications and the corresponding citation frequency and centrality. Among them, China published the most papers (N=193), followed by USA (N=164) and Germany (N=60), and the highest citation frequency was USA (N=15679), followed by China (N=7463) and Germany (N=4297). The average number of citations of Israel, England, and USA topped the list, and although China published the most papers, the average number of citations was lower than most countries/regions. **Figure 3** shows that research is mainly concentrated in the Northern Hemisphere, and it is worth noting that the links between countries/regions are mainly between North America and East Asia, and that Oceania is also relatively strongly linked to North America and East Asia. The total link strength of countries/regions measures the importance of the country/region’s position in the network. In summary, despite the large number of papers published in this area, USA’s research maintains its dominant position; and the number of articles published by Israel, although small, is on the whole of a high academic standard. **Supplementary Figure 1** illustrates the distribution of corresponding authors’ countries based on the number of documents, distinguishing between Single Country Publications (SCP) and Multiple Country Publications (MCP). China, the USA, and Germany are the leading countries in terms of total publications, with China having a higher number of Single Country Publications (SCP), while the USA shows a notable presence of Multiple Country Publications (MCP). Other countries like Italy, Japan, and Canada also contribute significantly to the research output. This chart highlights the global collaboration in the field, with a considerable portion of publications involving multiple countries.

**Table 1:** Top 10 countries/regions in terms of number of publications, the total link strength, the corresponding frequency of citations and average citations.

Rank	Countries/regions	Publications	Total Link Strength	Citations	Citations(av)
1	China	193	45	7463	38.66
2	USA	164	109	15679	95.6
3	Germany	60	54	4297	71.61
4	Italy	48	26	3126	65.125
5	Japan	38	12	1170	30.78
6	Israel	29	27	3341	115.206
7	France	22	17	1156	52.54
8	England	20	34	1979	98.85
9	Poland	20	7	702	35.1
10	Canada	19	21	1049	55.21

### 3.3 Distribution of institutions

**Figure 4:**
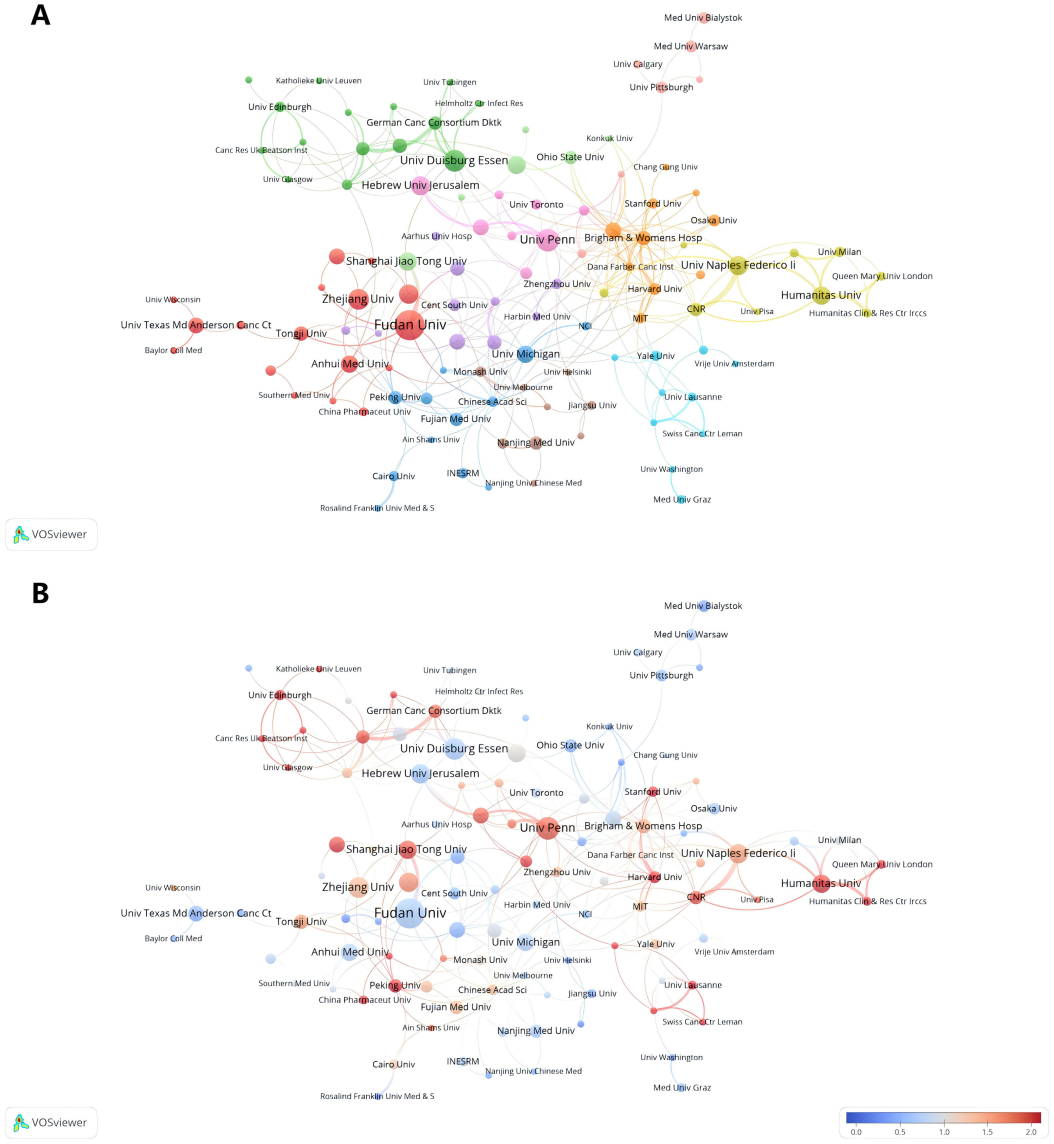
Analysis of TANs-related institution. **(A)** Analysis of collaborative network visualization of institutions in VOSviewer. The figure shows the institutions with more than 5 documents. The nodes of different colors represent the institutions of different clusters, and the size of the nodes indicates the frequency of their occurrence. **(B)** Analysis of the number of articles published by institutions in recent years. The recent 5 years heat value of each institution is obtained by dividing the number of publications in recent 5 year by the total number of publications.

**Table 2** lists the top 10 institutions in terms of number of publications, frequency of citations, and corresponding centrality. The top three institutions with the highest number of publications are: Fudan Univ (N=22), Univ Penn (N=15) and Univ Duisburg Essen (N=14). Among the top ten most productive institutions, 50% belonged to China, followed by two in Italy and one each in USA, Germany and Israel. The three institutions with the highest citation frequency are Univ Penn (N=4860), Stanford Univ (N=2096), and Harvard Univ (N=1929). It is worth noting that Brigham & Womens Hosp, Harvard Med Sch, Fudan Univ, and Univ Naples Federico-II showed a high total link strength, indicating that these institutions have a more important position in the research in the field of TANs, and may be the key nodes in the research field of TANs. Taken together Univ Penn has a much higher citation frequency, number of publications, and relatively high total link strength, which means that its research work on TANs has high visibility and influence in the academic community. Research institutions were analyzed to understand the global distribution of research related to TANs and to provide opportunities for collaboration. In VOSviewer, institutional collaborations are categorized into 11 closely related blocks (**Figure 4A**). **Figure 4B** shows the ratio of institutional publications to total publications obtained by dividing the number of TANs-related papers published by each institution in the last five years by the total number of papers published by each institution from 2007 to 2024, i.e., the ratio of institutional publications to total publications in the last five years. The color bias toward red means a high ratio, indicating that these institutions are emerging forces in the field of networking; the color bias toward blue means a low ratio, indicating that these institutions have done relatively little research in the field of TANs in recent years. The results show that the number of studies conducted by institutions such as Univ Penn, Humanitas Univ, and Shanghai Jiao Tong Univ has increased significantly over the past five years. In contrast, institutions such as Fudan Univ, Univ Duisburg Essen, and Hebrew Univ Jerusalem conducted relatively few studies in the past five years. Ranking the research institutions by Total Link Strength, **Table 2** also shows the top ten institutions with the highest Total Link Strength. compared to the top ten most prolific institutions, USA has a significant increase in the number of institutions with a total of five institutions on the list. three institutions from Italy are also ranked in the list, and one institution from China made the list.

**Table 2:** Top 10 institutions in terms of number of articles issued and top 10 institutions in terms of the total link strength.

Rank	Institution	Publications	Total Link Strength	citations	Institution	Country	Total Link Strength	citations
1	Fudan Univ	22	21	1288	Brigham & Womens Hosp	USA	30	1403
2	Univ Penn	15	18	4860	Harvard Med Sch	USA	26	594
3	Univ Duisburg Essen	14	15	633	Fudan Univ	China	21	1288
4	Zhejiang Univ	13	5	177	Univ Naples Federico Ii	Italy	21	903
5	Hebrew Univ Jerusalem	12	9	686	German Canc Res Ctr	Germany	18	403
6	Sun Yat Sen Univ	12	4	481	Humanitas Univ	Italy	18	1127
7	Univ Naples Federico Ii	12	21	903	Univ Penn	USA	18	4860
8	Huazhong Univ Sci & Technol	11	5	382	CNR	Italy	17	545
9	Humanitas Univ	11	18	1127	Dana Farber Canc Inst	USA	17	509
10	Shanghai Jiao Tong Univ	11	10	413	Harvard Univ	USA	15	1929

### 3.4 Distribution of authors

**Figure 5:**
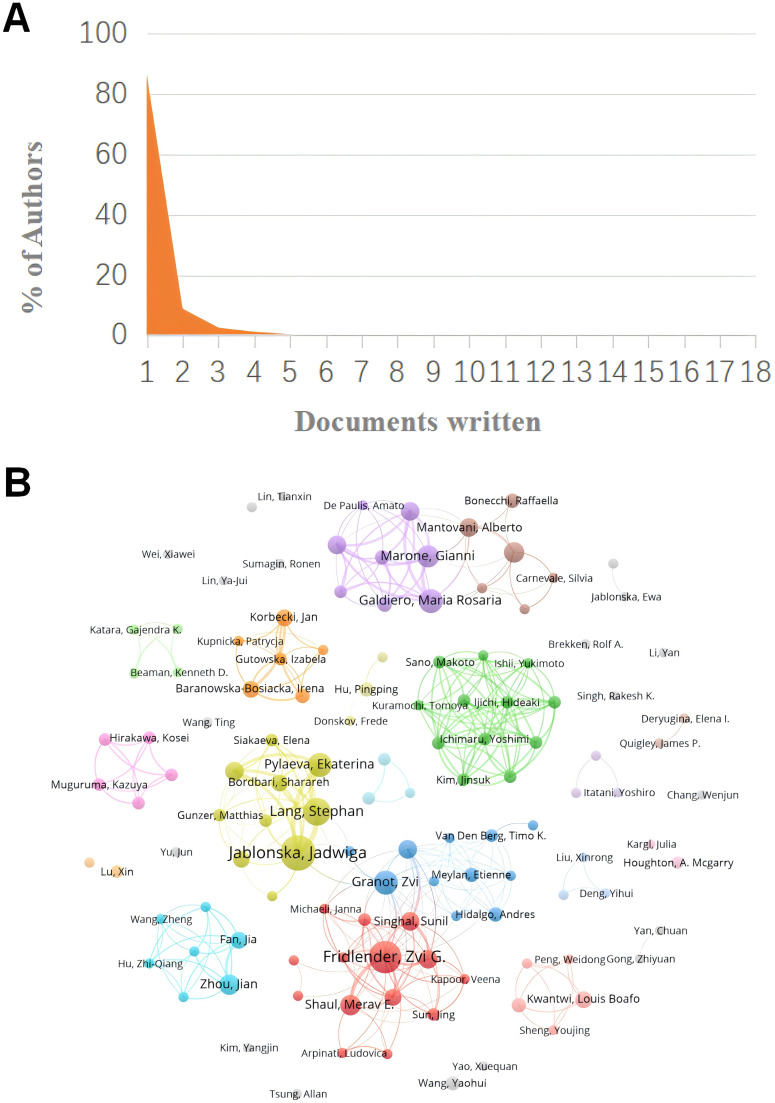
**(A)** Scientific productivity of authors based on Lotka’s Law. **(B)** The network map of authors on tumor-associated neutrophil research. The figure shows the authors with more than 2 documents.

A total of 3763 authors were involved in the study of tumor-associated neutrophils. Scientific productivity based on Lotka’s law showed that 86% of the authors published only one paper (**Figure 5A**) (40). The author with the most publications was Jablonska, Jadwiga (University of Duisburg Essen) (N=18), followed by Fridlender, Zvi G. (Univ Jerusalem) (N=16), Galdiero, Maria Rosaria (University of Naples Federico II) (N=11) and Granot, Zvi (Hebrew University of Jerusalem) (N=10). VOSvivewer shows collaboration between authors of literature related to TANs (**Figure 5B**), which provides the opportunity for researchers to find research partners in their own research field and to identify research partners and industry authorities in the field. Granot, Zvi and Marone, Ginanni the central figures of this collaborative network. As we can see from the **Figure 5B**, Granot, Zvi is associated with Fridlender, Zvi G., Jablonska, Jadwiga, and Marone, Ginanni is actively collaborating with Mantovani, Alberto, but other than that the clusters are relatively independent of each other and not closely connected, and this relatively decentralized collaborative network may indicate that authors in the field of TANs have been working together for many years. Network may indicate that there is less cross-national and cross-institutional co-research among authors in the field of TANs, or it may signal that the field of TANs has not yet gained widespread research. The co-cited author analysis refers to two authors whose literature is simultaneously cited by a third author (41). A higher citation frequency indicates a higher degree of consistency between these authors in terms of academic interest and depth of research. By analyzing the authors with the highest number of publications and co-citation frequency, the research strength of the authors and the research hotspots related to TANs can be visualized. **Table 3** gives the top 10 authors in terms of the number of publications 、citations and co-citation frequency, respectively. The most cited author is FRIDLENDER ZG (Univ Jerusalem) (N=4807), followed by ALBELDA SM (University of Pennsylvania) (N=3637), and the author with the highest co-citation frequency is Fridlender, Zvi G. (Univ Jerusalem) (N=540), followed by Mantovani, A (Humanitas University) (N=289). It is noteworthy that Fridlender, Zvi G. has a high impact in this field both in terms of citations and co-citations. The H-index, G-index and M-index are measures of the academic impact of a researcher, an academic journal or an institution. According to the H-index, papers submitted by an author or country/region are cited no less than H times but no more than H times. The key of the H-index is that it takes into account both the number of papers and the number of citations, which can reflect a scholar’s academic influence more comprehensively (43). The G-index helps to identify the highly cited papers of scholars, and thus reflects the scholar’s academic achievements more accurately (44). The M-index is mainly used to comprea the academic influence of different scholars in the same field, especially when the distribution of citation counts is similar (45). Combining the three indices, FRIDLENDER ZG and JABLONSKA J are two scholars with high academic influence in the field of TANs, and the two scholars are from Israel and Germany, respectively. Notably, three of the scholars with H-index in the top ten are from Israel, three from Italy, two from Germany, and one each from China and the United States.

**Table 3:** Top 10 cited authors and co-citated authors in the field of tumor-associated neutrophil, top 10 H-index authors in the field of tumor-associated neutrophil.

Rank	Author	Publications	Citations	Author	Publications	Co- citations	Total link strength	Author	H-index	G-index	M-index	Country
1	FRIDLENDER ZG	16	4807	FRIDLENDER ZG	16	540	4803	FRIDLENDER ZG	15	16	0.938	Israeli
2	ALBELDA SM	7	3637	MANTOVANI A	17	289	4023	JABLONSKA J	14	18	1.273	Germany
3	SUN J	5	2631	COFFELT SB	1	284	3755	GRANOT Z	10	10	0.909	Israeli
4	KAPOOR V	3	2610	SHAUL ME	8	247	3784	GALDIERO MR	9	11	0.9	Italy
5	CHENG GJ	2	2460	ERUSLANOV EB	4	221	3515	LANG S	8	10	0.889	Germany
6	WORTHEN GS	2	2460	GRANOT Z	10	211	2809	PYLAEVA E	8	10	0.889	Germany
7	KIM S	1	2220	MISHALIAN I	6	178	3835	MARONE G	8	9	0.889	Italy
8	LING LN	1	2220	GABRILOVICH DI	1	177	3500	SHAUL ME	8	8	0.889	Israeli
9	XU Y	2	2065	ANDZINSKI L	3	167	2946	ZHOU J	7	8	0.778	China
10	NAIR VS	2	2032	JABLONSKA J	18	164	1951	ALBELDA SM	7	7	0.438	USA

### 3.5 Journal publication analysis

**Figure 6:**
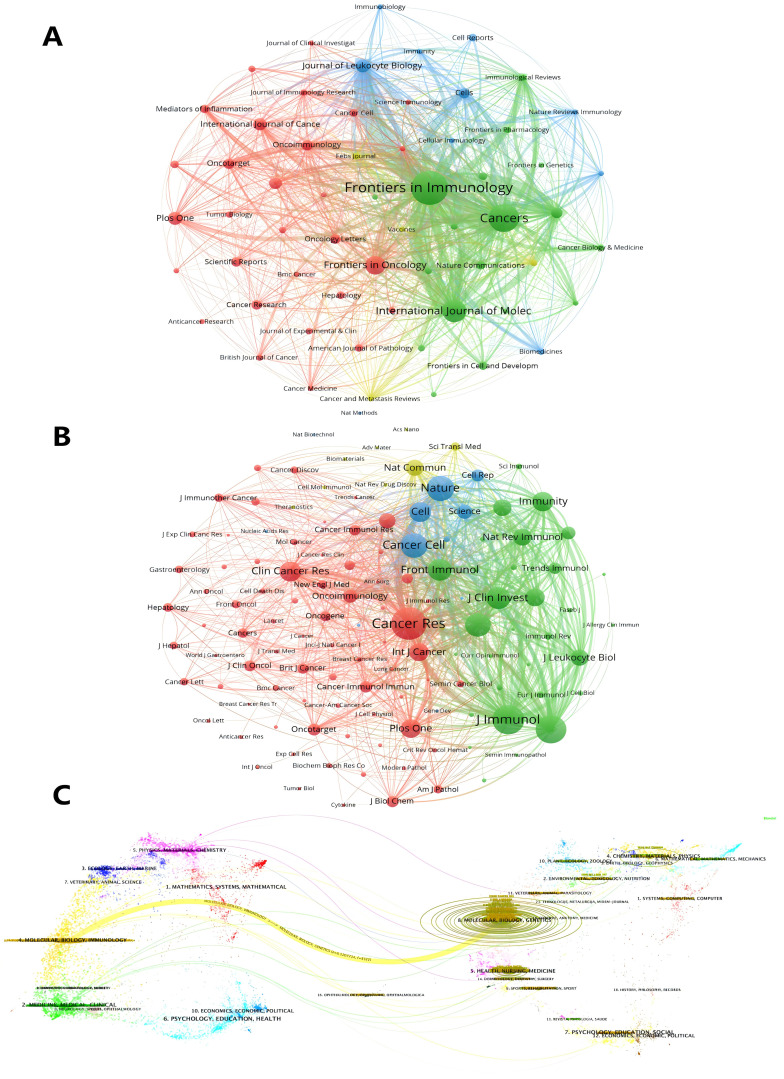
Analysis of TANs-related journal. **(A)** Analysis of collaborative network visualization of journals in VOSviewer. The figure shows the journals with more than 10 documents. The nodes in different colors represent the journals in different clusters, and the size of the nodes indicates the frequency of their occurrence. **(B)** Analysis of collaborative network visualization of journals’ citations in VOSviewer. The size of the nodes indicates the frequency of their occurrence. The figure shows the journals with more than 75 citations. **(C)** The dual-map overlay of journals. Citing journals are on the left, cited journals are on the right, and colored paths indicate citation relationships.

Journals ranked in the top 25% (inclusive) of the impact factor are located in JCR quartile 1 (Q1), and journals ranked in the top 25%-50% (inclusive) of the impact factor are located in JCR quartile 2 (Q2). **Table 4** lists the top 10 journals in terms of number of articles and their corresponding IF (JCR2023). The journal with the highest number of publications was Frontiers in Immunology (5. 7, Q1) (43), followed by CANCERS (4. 5, Q1) (32), INTERNATIONAL JOURNAL OF MOLECULAR SCIENCES (4. 9, Q1) (23) and FRONTIERS IN ONCOLOGY (3. 5, Q2) (18). There are 12 journals in the top ten in terms of publications, seven journals distributed in the Q1 JCR, and only five journals with an IF of 5 or more. Among these journals, the most frequently cited journals are Frontiers in Immunology, Cancers and International Journal of Molecular Sciences. SEMINARS IN IMMUNOLOGY had the highest impact factor (IF=7. 4), followed by ONCOIMMUNOLOGY(IF=6. 5). It is worth noting that ONCOTARGET, despite having a large number of publications, has not been indexed by SCI since 2018, so the papers retrieved from this journal are all before 2018, which may mean that the journal fails to meet the appropriate academic standards or quality requirements, leading to a decline in academic recognition of the research results published in this journal. 2023 JOURNAL Most of the top 10 co-cited journals in the Citation Report (JCR) are located in the Q1 region, with the exception of Journal of Immunology. The impact of academic journals depends on the number of times they are co-cited, which indicates their importance in a particular research area (**Table 4**). The journals with the highest co-citation frequency were Cancer Res (12. 5, Q1) (2182) and Journal of Immunology (3. 6, Q2) (1888). Nine of the top 10 journals in terms of co-citation frequency are distributed in JCR Q1, and seven journals have an IF of more than 10. The visualization map generated by VOSviewer shows the various types of journals involved in research in the field of TANs and their interconnections with each other. These journals are grouped into different clusters based on the similarities between them (**Figure 6A**), and are generally divided into 4 categories: The blue cluster focuses on research in autoimmunity and cell biology (Journal of Leukocyte Biology, Immunobiology, Cells, etc.); the green cluster focuses on research in immunity and cancer (Frontiers in Immunology, Cancers, International Journal of Molec, etc.); the red cluster focuses on oncology (Frontiers in Oncology, Oncoimmunology, etc.); and the yellow cluster focuses on clinical research and treatment and molecular biology related fields (Febs Journal, Cancer and Metastasis Reviews, etc.). Based on the co-citation frequency, these journals were categorized into four groups, which tended to have similar research directions (**Figure 6B**). The red cluster focuses on cancer-related fields (Cancer Research, Clinical Cancer Research, etc.); the green cluster focuses on immunology (Frontiers in Immunology, Journal of Clinical Investigation, etc.); and the blue cluster is mainly in the biochemistry and molecular biology (Nature, Cell, Science, etc.); the yellow cluster is mainly in the field of translational medicine (Nature Communications, Science Translational Medicine, etc.). **Supplementary Figure 1** presents the annual heat map of journals for the past decade. The data can be roughly divided into three modules. In 2015-2016, the most highly cited journals were concentrated in the following areas: Seminar in Immunology (IF=7. 4), Cancer Cell (IF=48. 8), and International Journal of Cancer (IF=5. 7). All three belong to the journals in JCR region 1, which have a high impact factor. In the 2017-2018 period, the focus was on the JOURNAL OF LEUKOCYTE BIOLOGY (IF=3. 6), PLOS ONE (IF=2. 9), and SCIENTIFIC REPORTS (IF=3. 8), which have relatively low impact factors. Subsequently, in the period following 2019, the focus shifts back to high-impact factor journals such as Nature Communications (IF=14. 7), Cancers (IF=4. 5) and Cells (IF=5. 1). We used knowledge flow analysis to explore the evolution of knowledge citations and co-citations among cited journals (**Figure 6C**), the journal bilabeled graphs show the thematic distribution of scholarly journals, changes in citation trajectories and changes in research centers, with the labels on the left representing Citing journals, and the labels on the right representing the cited journals (46, 47). The citation linkage of the colored curves pointing from the citation graph to the cited graph show the citation connections. Citing journals are mainly from the fields known as research frontiers, such as MOLECULAR, BLOLOGY, and IMMUNOLOGY. The cited journals are mainly from the fields known as knowledge bases. It is worth noting that both Citing journals, and the cited journals belong to the same label, which suggests that research on TANs is still concentrated in certain areas and has not expanded to other areas.

**Table 4:** Top 10 journals in terms of number of publications, corresponding IF (JCR 2023) and JCR quartile.

Rank	Journal	Publications	IF(JCR 2023)	Journal	IF(JCR 2023)	Citations	Co- citations
1	FRONTIERS IN IMMUNOLOGY	42	5.7	Cancer Res	12.5	793	2182
2	CANCERS	32	4.5	Journal of Immunology	3.6	365	1888
3	INTERNATIONAL JOURNAL OF MOLECULAR SCIENCES	23	4.9	Blood	21	353	1767
4	FRONTIERS IN ONCOLOGY	18	3.5	Nature	50.5	145	1546
5	JOURNAL OF LEUKOCYTE BIOLOGY	11	3.6	Cancer Cell	48.8	2844	1467
6	PLOS ONE	11	2.9	Proceedings of the National Academy of Sciences of the United States of America	9.4	801	1380
7	INTERNATIONAL JOURNAL OF CANCER	10	5.7	Journal of Clinical Investigation	13.3	438	1268
8	CANCER IMMUNOLOGY IMMUNOTHERAPY	9	4.6	Frontiers in Immunology	5.7	2247	1242
9	CELLS	8	5.1	Clinical Cancer Research	10	371	1087
10	ONCOIMMUNOLOGY	8	6.5	Cell	45.5	14	1079
10	ONCOTARGET	8	5.168(2016)	—	—	—	—
10	SEMINARS IN IMMUNOLOGY	8	7.4	—	—	—	—

### 3.6 Keyword analysis

**Figure 7:**
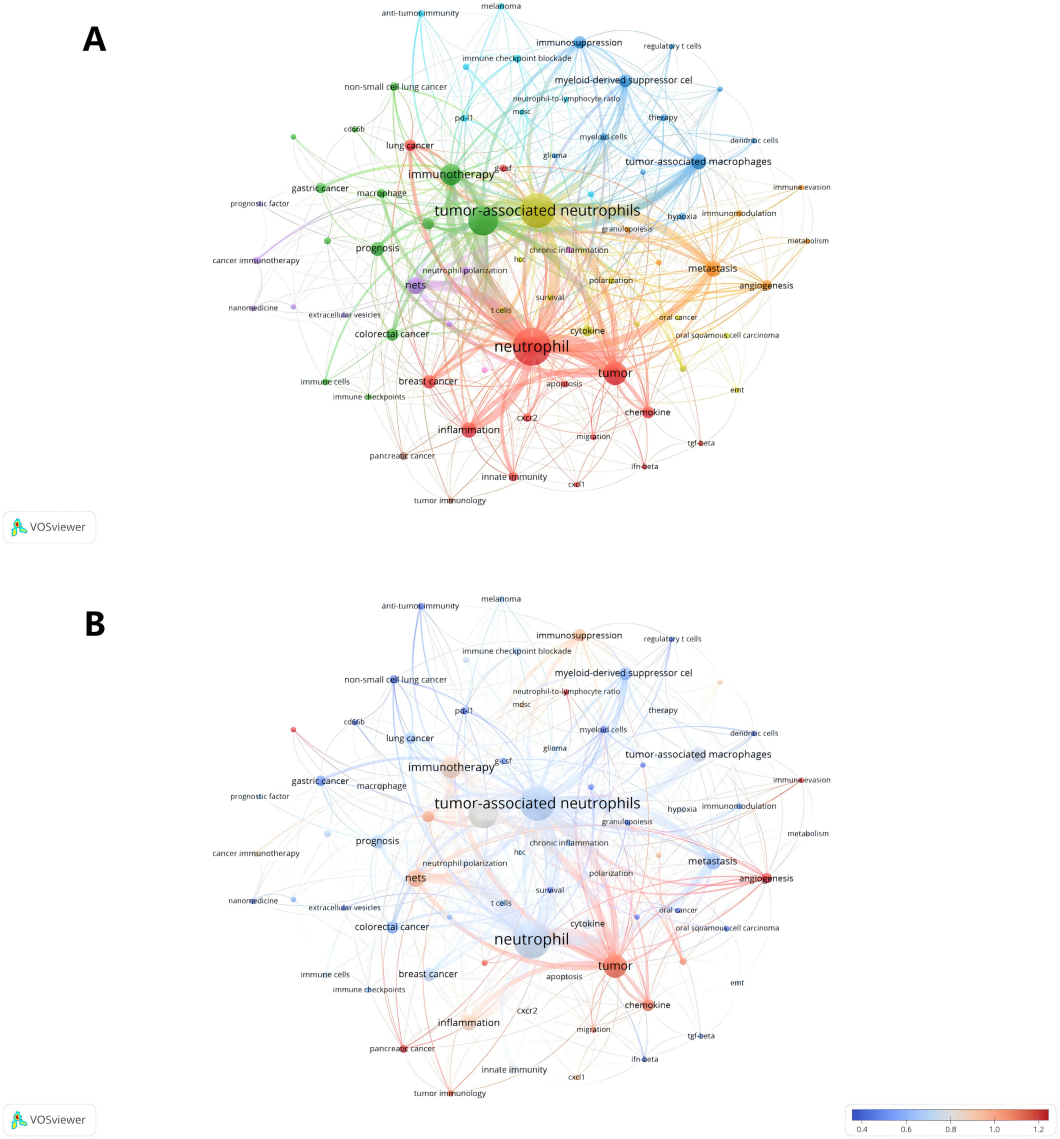
Analysis of TANs-related keyword. **(A)** The network map of keywords on tumor-associated neutrophil research. The figure shows the keywords with more than 4 occurrences. **(B)** A keyword impact analysis. Represents the avg-normal-citations for the article in which the keyword is found.

**Figure 8:**
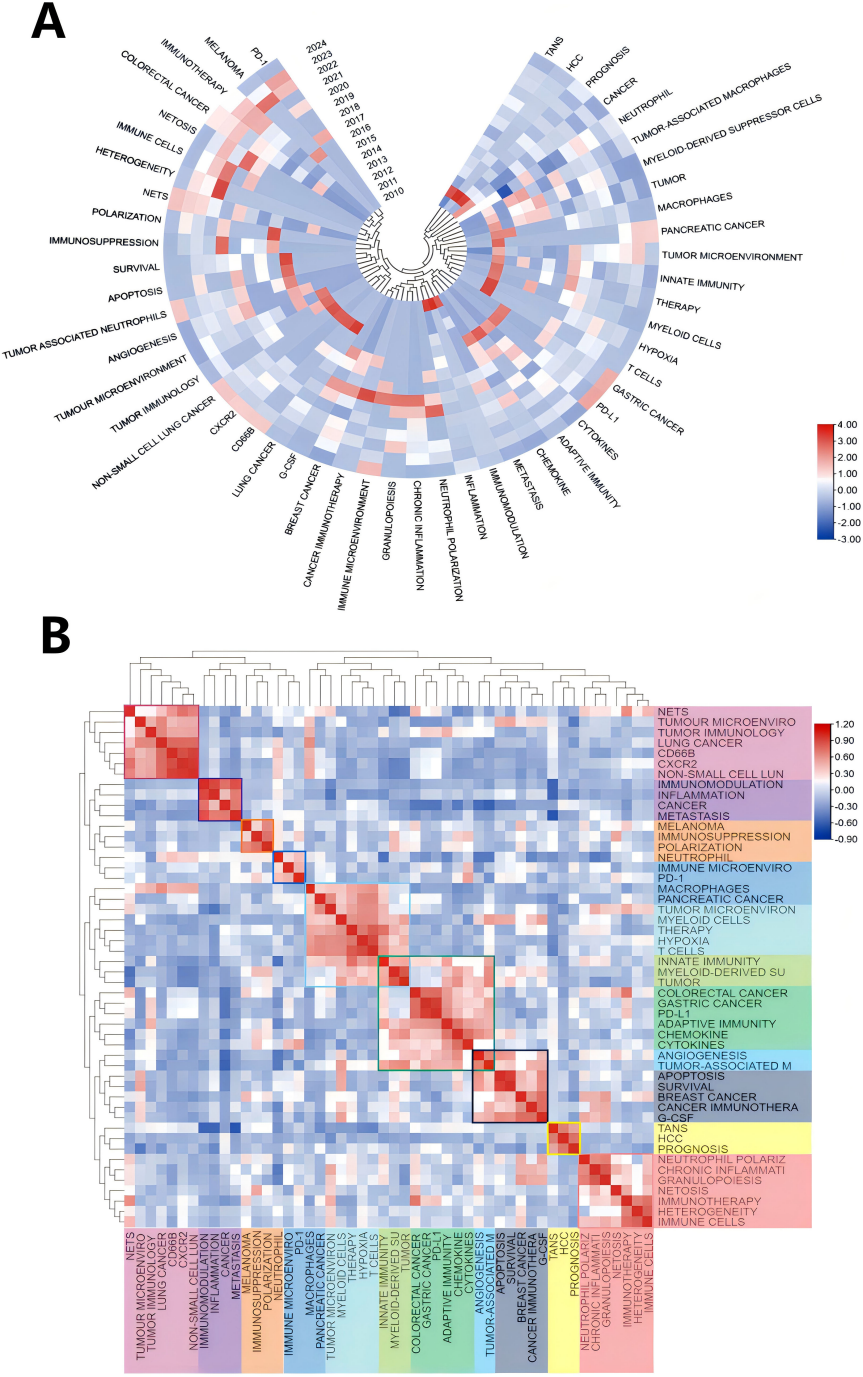
**(A)** Annual heatmap from 2010 to 2024, where the annual heat value of each keyword is obtained by dividing the number of citations in that year by the total number of citations in that year, with the top 50 keywords based on occurrences. **(B)** Keyword relevance heatmap. Keywords with high popularity in similar time periods are clustered into one category and marked with different colors.

Keywords play a crucial role in academic papers, as they concisely summarize the core topic, objectives, target audience, and methodology employed in the research. A systematic analysis of keywords can reveal the trends and evolution of research in a particular academic field, as well as the focus of research at a given time (48). Keywords are not only a quick way to understand the main idea of a paper, but also an important indicator of the concerns and research hotspots in an academic field (48). **Table 5** shows the top 20 keywords in order of frequency. The most frequent keyword is “neutrophil” (179), followed by “tumor-associated neutrophils” (49). In addition, “tumor microenvironment” (50) and “tumor” (51) are frequently occurring keywords, indicating that their corresponding fields are popular in TANs-related research. **Table 5** also shows the specific diseases appearing in the research field of TANs. breast cancer and colorectal cancer appeared more than 20 times, among which breast cancer had the highest number of occurrences and the highest Total link strength, and it is worth noting that lung cancer, which appeared less than 20 times, had a higher Total link strength. We used VOSviewer software to extract 76 keywords from the titles and abstracts of 615 articles, and set the minimum number of citations to 4, resulting in a total of 60 keywords. We drew a visualization map of these 60 keywords (**Figure 7A**) and found that all the keywords were roughly classified into 7 major categories, representing 7 different research directions in the field of TANs: the red clusters were related to inflammatory response and tumor development, the green clusters were related to tumor immunity, the purple clusters were mainly related to immune regulation, the blue clusters were related to the immune-suppressing mechanisms in the tumor microenvironment, the cyan clusters were related to the tumor immunotherapy, yellow clustering is related to the role of TANs in tumors, and orange clustering is related to tumor metastasis, angiogenesis and metabolic regulation mechanisms. **Figure 7B** shows the impact analysis of keywords, represented by the average number of normal citations for articles with a given keyword. A redder keyword represents a hotter keyword, which means that articles with this keyword have a high average number of citations; a bluish color is the opposite. Analysis of the results of this graph shows that the terms pancreatic cancer, tumor, chemokine, angiogenesis, immune evasion, neutrophil-to-lymphocyte ratio, etc. are the most popular keywords. Notably, these keywords are all related to immune escape from tumors in some way, which implies that more and more studies tend to explore the tumor-promoting functions of TANs. inflammation, cxcl1, nets, immunosuppression, neutrophil, prognosis, T cells therapy, glioma, etc. are followed by the research heat, and these keywords reflect the attention to the inflammatory response, immune regulation, tumor biology, and related therapeutic approaches in the field of TANs research, revealing their research hotspots in pathophysiological mechanisms, disease progression, and therapeutic strategies. **Figure 8A** shows the annual popularity (annual citations/total annual citations) of the keywords from 2007 - 2024. The annual popularity of the keywords HCC, MYELOID-DERIVED SUPPRESSOR CELLS, and IMMUNOMODULATION has been relatively low in recent years. In contrast, the annual popularity of keywords such as NETs, COLORECTAL CANCER, IMMUNOTHERAPY, and HETEROGENEITY has been relatively high in recent years, indicating that these keywords represent emerging frontiers. **Figure 8B** shows the keyword popularity correlation, where keywords with high popularity in similar time periods are clustered in different clusters and marked with different colors. The results show nine clusters: Pink Clusters (NETS, TUMOUR MICROENVIRO, TUMOR IMMUNOLOGY, CANCER, CD66B, CXCR2, NON-SMALL CELL LUN), Purple Clusters (IMMUNOMODULATION, INFLAMMATION, CANCER METASTASIS), Orange Cluster (MELANOMA, IMMUNOSUPPRESSION, POLARIZATION), Blue Cluster (NEUTROPHIL, IMMUNE MICROENVIRO, PD-1), Cyan Cluster (MACROPHAGES, PANCREATIC CANCER, TUMOR MICROENVIRON, MYELOID CELLS, THERAPY, HYPOXIA, T CELLS, INNATE IMMUNITY, MYELOID-DERIVED SU, TUMOR), Green Clusters (INNATE IMMUNITY, MYELOID-DERIVED SU TUMOR, COLORECTAL CANCER, GASTRIC CANCER, PD-L1, ADAPTIVE IMMUNITY, CHEMOKINE, CYTOKINES, ANGIOGENESIS, TUMOR-ASSOCIATED M), Dark Blue Clusters (ANGIOGENESIS TUMOR-ASSOCIATED M, APOPTOSIS, SURVIVAL, BREAST CANCER, CANCER IMMUNOTHERA, G-CSF), Yellow Cluster (TANS, HCC, PROGNOSIS), Red Cluster (NEUTROPHIL POLARIZ, CHRONIC INFLAMMATI, GRANULOPOIESIS, NETOSIS, IMMUNOTHERAPY, HETEROGENEITY, IMMUNE CELLS). The areas where Cyan and Green overlap are filled with Light Green, and the areas where Green and Dark Blue overlap are filled with Light Blue. This indicates that the keywords within the same cluster have high popularity within the same time period. It is worth noting that the keywords in the green cluster partially intersect with the keywords in the cyan and dark blue clusters, respectively, which we have labeled in dark red in the figure. These keywords are INNATE IMMUNITY, MYELOID-DERIVED SU, TUMOR, ANGIOGENESIS, and TUMOR-ASSOCIATED M. These five keywords have high prevalence in multiple time periods, suggesting that they are more favored by researchers.

**Table 5:** Top10 keywords in terms of frequency of occurrence and the corresponding total link strength and top 10 disease related to tumor associated neutrophil research.

Rank	Keyword	Occurrences	Total link strength	Keyword	Occurrences	Total link strength
1	neutrophil	179	394	breast cancer	24	56
2	tumor-associated neutrophils	151	342	colorectal cancer	21	40
3	tumor microenvironment	113	292	hepatocellular carcinoma	18	43
4	tumor	70	194	lung cancer	18	50
5	immunotherapy	58	145	gastric cancer	15	25
6	nets	38	99	non-small cell lung cancer	10	26
7	metastasis	34	109	pancreatic cancer	9	19
8	inflammation	32	77	chronic inflammation	6	16
9	tumor-associated macrophages	32	107	melanoma	6	16
10	prognosis	25	50	oral squamous cell carcinoma	5	8

**Supplementary Figure 2** illustrates the relationships between various research fields, with the size of each node representing the prominence of the field. “Oncology” stands out as the most central and influential field in this network, indicating its significant role in the broader research landscape. Other fields, such as “Immunology,” “Biochemistry & Molecular Biology,” “Gastroenterology & Hepatology,” and “Pharmacology & Pharmacy,” show strong connections, highlighting the interdisciplinary nature of research in these areas. Interestingly, the relatively small fields of “Nanoscience & Nanotechnology” and “Biophysics” have high centrality, representing the bridging role of these fields.

### 3.7 Highly cited reference analysis

**Figure 9:**
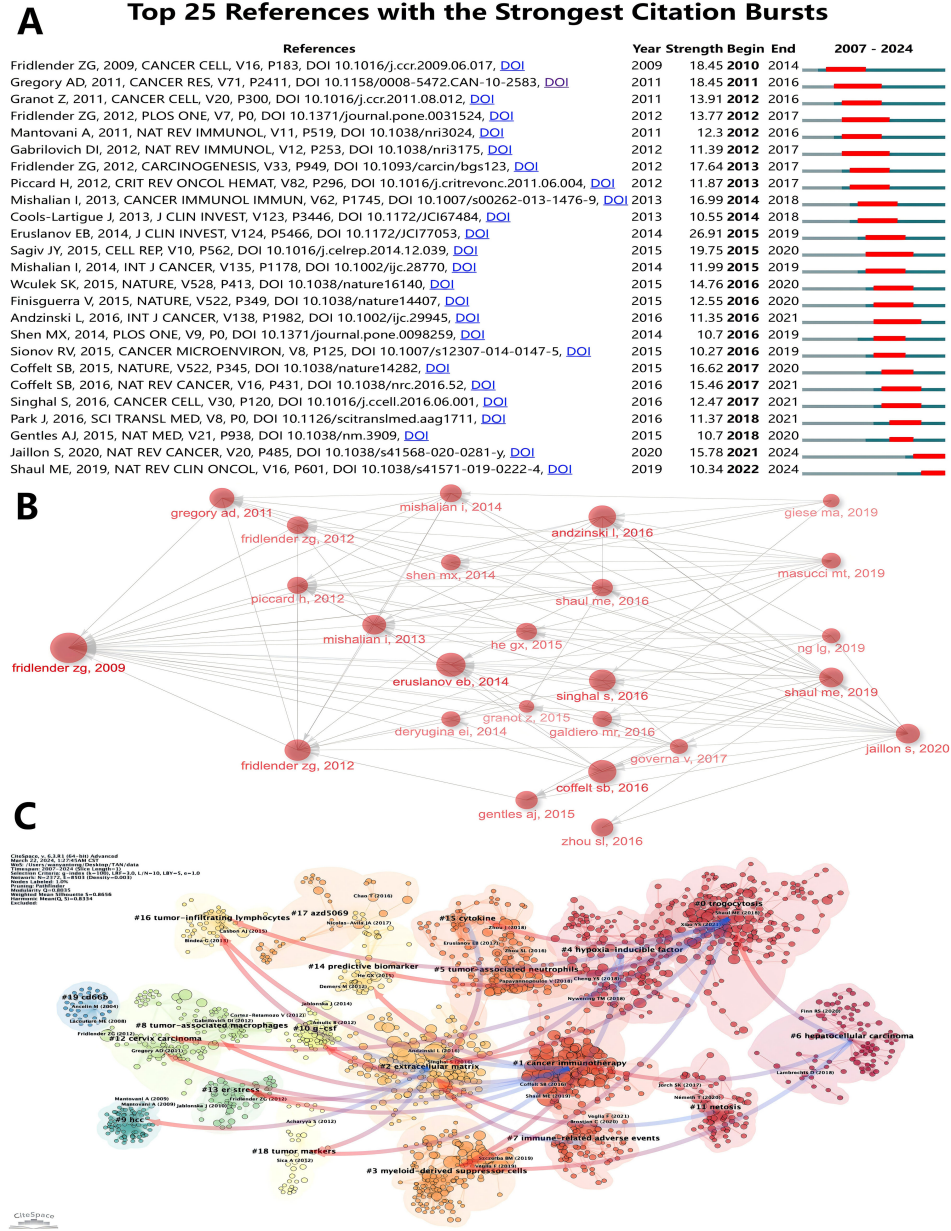
**(A)** Top 25 References with the Strongest Citation Bursts. **(B)** Association between the top 25 highly cited documents. **(C)** Clustering of references based on similarity between references, with a threshold of k=100, using Pathfinder and pruning sliced networks.

**Table 6** lists the top ten most frequently cited articles. The most frequently cited article is “Polarization of Tumor-Associated Neutrophil Phenotype by TGF-*β*: N1 versus N2 TANs” (Fridlender, ZG, et al., 2009) (2220) (7) (39), which focuses on how TGF-*β* blockers alter the phenotype of tumor-associated neutrophils (TANs) from a pro-tumorigenic “N2” phenotype to an anti-tumorigenic “N1” phenotype, which slows tumor growth and enhance the activation of CD8+ T cells. It is noteworthy that this is the first paper in the field to present a detailed concept and systematic study of TANs, which has laid the foundation for its high citation rate. Next, “The prognostic landscape of genes and infiltrating immune cells across human cancers” (Gentles, AJ, et al., 2015) (2025) (52), which is also the article with the highest average citation frequency per year, this paper reveals the prognostic landscape of genes and tumor-infiltrating immune cells across different types of cancers by comprehensively analyzing the expression profile and overall survival data of approximately 18, 000 human tumors, identifying the FOXM1 regulatory network as a major predictor of poor prognosis, while pointing out that genes such as KLRB1 expression is associated with positive prognosis in tumor-associated leukocytes, which includes tumor-associated neutrophils. **Figure 9A** shows the top 25 references with the strongest citation bursts. The first two citation bursts occurred in 2010 and 2011. They are titled “Polarization of Tumor-Associated Neutrophil Phenotype by TGF-*β*: N1 versus N2 TANs” and “Tumor-associated Neutrophils: New Targets for Cancer Therapy”. Associated Neutrophils: New Targets for Cancer Therapy”. Notably, “Tumor-associated neutrophils stimulate T cell responses in early-stage human lung cancer” was published in The Journal of Clinical Investigation by Eruslanov EB et al. in 2014. al in The Journal of Clinical Investigation in 2014 (Strength= 26. 91), and its outbreak lasted from 2014 to 2019. Sagiv JY’s “Hyperglycemia Impairs Neutrophil Mobilization Leading to Enhanced Metastatic Seeding” published by Sagiv JY in Cell Reports also had a high outbreak (Strength= 19. 75). The results showed that 2016 had the highest number of newly cited outbreaks (5), followed by 2012 (4), indicating that the high number of outbreaks in these two years triggered a related research boom. Article co-citation analysis analyzed the relationship between articles by analyzing their co-citation frequency. Relationships between studies were displayed in CiteSpace, and the authors and years of the 25 most frequently cited articles are shown in **Figure 9B**. The results show that Fridlender, ZG et al. (7) in 2009 published “Polarization of Tumor-Associated Neutrophil Phenotype by TGF-*β*: N1 versus N2 TANs’ was the most cited, which may have some relevance to its earliest outbreaks. This was followed by the most cited articles from 2014 (4) and 2016 (6), which served as links. Ultimately, the majority of articles were cited in “Neutrophil diversity and plasticity in tumor progression and therapy” by Jaillons et al., 2020 (55). In bibliometric studies, the analysis of Local Citations can help us to understand the research dynamics of a specific field or discipline, identify core authors and key literature, as well as assess the scholarly contribution of a research organization or an individual. **Supplementary Figure 2** shows the ten articles with the highest Local Citations in WOSCC. It is worth noting that the Fridlender, ZG et al. published in 2009 are again at the top and three of them are from 2016. The clustering is based on the degree of association between the literature and is divided into 19 categories, indicated by different colors (**Figure 9C**). The category with the highest number of published articles is #0, and the most common keyword in these articles is trogocytosis. Chronologically, the earliest areas of research in TANs were stand-alone research clusters:#19 cd66b, and #9 hcc was also a relatively early research cluster, which developed into #2 extracellular matrix. #2 extracellular matrix then developed into #0 trogocytosis, #4 hypoxia-inducible factor, #7 immune-related adverse events, #11 netosis, #15 cytokine, and in addition, #17 azd5069 later became a stand-alone research cluster; #16 developed primarily into cluster #10, with #10 g-csf, #13 er stress, #16 tumor-infiltrating lymphocytes, and #18 tumor markers developing together into #1 cancer immunotherapy and #2 extracellular matrix clusters. 2 extracellular matrix clusters; #0 and #1 in turn developed together into #6. after 2012, #16 tumor-infiltrating lymphocytes and #18 tumor markers were closely related and developed into three relatively independent groups, #0 trogocytosis, #1 cancer immunotherapy, and #2 extracellular matrix. Subsequently, the closeness of the links between the study regions declined further, with the emergence of several relatively independent clusters, including #7 immune-related adverse events, #11 netosis, #17 azd5069.

**Table 6:** Top 10 highly cited article.

Rank	Author	Source Title	Article Title	Year	Cited	DOI
1	Fridlender, ZG; Sun, J; Kim, S; Kapoor, V; Cheng, GJ; Ling, LN; Worthen, GS; Albelda, SM (39)	CANCER CELL	Polarization of Tumor-Associated Neutrophil Phenotype by TGF-β: N1 versus N2 TANs	2009	2220	10.1016/j.ccr.2009.06.017
2	Gentles, AJ; Newman, AM; Liu, CL; Bratman, SV; Feng, WG; Kim, D; Nair, VS; Xu, Y; Khuong, A; Hoang, CD; Diehn, M; West, RB; Plevritis, SK; Alizadeh, AA (52)	NATURE MEDICINE	The prognostic landscape of genes and infiltrating immune cells across human cancers	2015	2025	10.1038/nm.3909
3	Coffelt, SB; Wellenstein, MD; de Visser, KE	NATURE REVIEWS CANCER	Neutrophils in cancer: neutral no more	2016	1102	10.1038/nrc.2016.52
4	Mayadas, TN; Cullere, X; Lowell, CA	ANNUAL REVIEW OF PATHOLOGY: MECHANISMS OF DISEASE, VOL 9	The Multifaceted Functions of Neutrophils	2014	816	10.1146/annurev-pathol-020712-164023
5	Xue, JW; Zhao, ZK; Zhang, L; Xue, LJ; Shen, SY; Wen, YJ; Wei, ZY; Wang, L; Kong, LY; Sun, HB; Ping, QN; Mo, R; Zhang, C	NATURE NANOTECHNOLOGY	Neutrophil-mediated anticancer drug delivery for suppression of postoperative malignant glioma recurrence	2017	607	10.1038/NNANO.2017.54
6	Kim, J; Bae, JS	MEDIATORS OF INFLAMMATION	Tumor-Associated Macrophages and Neutrophils in Tumor Microenvironment	2016	545	10.1155/2016/6058147
7	Gregory, AD; Houghton, AM	CANCER RESEARCH	Tumor-Associated Neutrophils: New Targets for Cancer Therapy	2011	526	10.1158/0008-5472.CAN-10-2583
8	Lazennec, G; Richmond, A	TRENDS IN MOLECULAR MEDICINE	Chemokines and chemokine receptors: new insights into cancer-related inflammation	2010	526	10.1016/j.molmed.2010.01.003
9	Zhou, SL; Zhou, ZJ; Hu, ZQ; Huang, XW; Wang, Z; Chen, EB; Fan, J; Cao, Y; Dai, Z; Zhou, J (53)	GASTROENTEROLOGY	Tumor-Associated Neutrophils Recruit Macrophages and T-Regulatory Cells to Promote Progression of Hepatocellular Carcinoma and ResisTANsce to Sorafenib	2016	515	10.1053/j.gastro.2016.02.040
10	Shaul, ME; Fridlender, ZG (54)	NATURE REVIEWS CLINICAL ONCOLOGY	Tumor-associated neutrophils in patients with cancer	2019	492	10.1038/s41571-019-0222-4

In scientometrics and network analysis, centrality serves as an indicator of the relative importance or influence of nodes within a network. A high centrality value signifies that a node (e.g., a document or author) holds significant connections or interactions with other nodes. Generally, nodes with high centrality play a key role in disseminating information, influencing decision-making, and shaping knowledge networks. These nodes, often regarded as ‘hubs,’ are central to the flow of information and can guide or promote the dissemination of knowledge. In academic literature networks, a high centrality score may indicate that a document has a substantial influence in a specific research area or that an author’s work has garnered widespread academic attention. In the field of tumor-associated neutrophils (TANs), three references stand out with centrality scores greater than 0.1, signaling their pivotal contributions to the development of the field and potential scientific breakthroughs (**Table 7**). The article “Tissue-infiltrating neutrophils constitute the major *in vivo* source of angiogenesis-inducing MMP-9 in the tumor microenvironment” has the highest centrality. This groundbreaking study first demonstrated that TANs, which can rapidly release pre-stored contents, are key contributors to the highly angiogenic MMP-9 in tumors. The second article, “Neutrophil function: from mechanisms to disease,” is a comprehensive review that systematically explores the role of neutrophils in diseases, particularly in tumors, establishing it as a leading work in the field. The third article, “TRPM2 Mediates Neutrophil Killing of Disseminated Tumor Cells,” identifies the mechanism by which neutrophils kill disseminated tumor cells in a Ca2+-dependent manner via TRPM2, shedding light on how neutrophils limit metastatic spread.

**Table 7:** Top 10 highly centrality references.

Rank	References	Count	Centrality	Year
1	Deryugina EI, 2014, NEOPLASIA, V16, P771, DOI 10.1016/j.neo.2014.08.013	20	0.13	2014
2	Amulic B, 2012, ANNU REV IMMUNOL, V30, P459, DOI 10.1146/annurev-immunol-020711-074942	9	0.12	2012
3	Gershkovitz M, 2018, CANCER RES, V78, P2680, DOI 10.1158/0008-5472.CAN-17-3614	38	0.12	2018
4	Zhang DC, 2015, NATURE, V525, P528, DOI 10.1038/nature15367	14	0.09	2015
5	Bierie B, 2006, NAT REV CANCER, V6, P506, DOI 10.1038/nrc1926	2	0.08	2006
6	Ardi VC, 2009, J BIOL CHEM, V284, P25854, DOI 10.1074/jbc.M109.033472	4	0.08	2009
7	Gabrilovich DI, 2009, NAT REV IMMUNOL, V9, P162, DOI 10.1038/nri2506	9	0.07	2009
8	Pahler J, 2008, NEOPLASIA, V10, P329, DOI 10.1593/neo.07871	2	0.06	2008
9	Dumitru CA, 2012, CANCER IMMUNOL IMMUN, V61, P1155, DOI 10.1007/s00262-012-1294-5	9	0.06	2012
10	Glodde N, 2017, IMMUNITY, V47, P789, DOI 10.1016/j.immuni.2017.09.012	26	0.06	2017

## 4 Discussion

Tumor-associated neutrophils (TANs) are integral to the tumor microenvironment, where they interact with tumor cells to sustain cancer properties and form a complex network of interactions between cancer cells and immune cells. To delineate the temporal and spatial distribution, key contributors, central publications, and to identify research hotspots and frontiers, we conducted an analysis of 615 neutrophil-related literature on tumors using the CiteSpace 6. 3. R2 Advanced, VOSviewer 1. 6. 18, and Rbibliometrix software. This analysis was based on data extracted from Web of Science for the period spanning from 2000 to 2024. The upward trend observed in the annual publication count underscores the significant potential of TANs in cancer research, indicating that this field is burgeoning and ripe for further exploration.

### 4.1 General information

The present study conducted an analysis of 615 TANs-related articles, which were sourced from the Web of Science Core Collection (WOSCC) database. These articles were authored by 3763 researchers affiliated with 934 institutions across 51 countries, and were published between January 1, 2000, and March 21, 2024. The exponential growth in the number of articles over this period highlights the escalating interest in TANs within the scientific community. The concept of TANs was formally introduced in 2009 by Fridlender ZG and colleagues, marking the inception of focused research in this domain. Since then, the field has seen a notable increase in scholarly output, particularly over the past decade. Notably, the number of publications in 2022 was approximately ten times that of 2012, a statistic that underscores the vibrant and burgeoning nature of TANs research in recent years.

In the national and regional analysis, the metrics of publication count and Total Link Strength are pivotal for evaluating a country or region’s role within the global research cooperative network. A higher Total Link Strength suggests that the country or region may serve as a hub within the research field. From a global perspective, as illustrated in **Table 1**, **Figure 3**, China and the United States emerge as the central research hubs in the network domain. China leads in the number of publications, with the second-highest citation frequency, while the United States follows closely with the second-highest publication count and the highest citation frequency. The United States also boasts the highest Total Link Strength of 109, outpacing all other nations, with Germany ranking second with a Total Link Strength of 54, signifying its significant role in global research collaboration. Furthermore, countries such as China, Italy, Israel, and the United Kingdom have actively engaged in network research and cooperation. Among the top 10 institutions, five are Chinese, and two are Italian. Notably, the University of Pennsylvania (Univ Penn) in the United States stands out with a significantly higher citation frequency compared to other institutions. Its high citation rate and Total Link Strength are indicative of the high quality of its research and its recognition within the academic community. Additionally, global research institutions have established cooperative networks, with emerging entities like Humanitas University and Shanghai Jiao Tong University showing a marked increase in their research output in the network field. This surge suggests that these institutions may be poised to become new leaders in the field. Conversely, institutions such as Fudan University and the University of Duisburg-Essen have exhibited a relatively lower research activity in recent years, which could imply a shift in their research focus or a commitment to more in-depth, long-term research endeavors. Based on the analysis, China and the United States play a pivotal role in the global network research landscape. China demonstrates its robust research output capacity, evidenced by its leading position in publication numbers. Meanwhile, the United States asserts its leading role and extensive influence in global research cooperation, as reflected in its high citation frequency and Total Link Strength. Furthermore, while Univ Penn in the United States is among the top research institutions, there is also a notable rise of new institutions that are making significant contributions to the field.

In the realm of author contributions, the seminal work by Jablonska, Jadwiga; Fridlender, Zvi G.; Galdiero, Maria Rosaria; Granot, Zvi and others indicate their pivotal role in the field of TANs. As depicted in **Table 3**, Fridlender, Zvi G., hailing from the University of Jerusalem, Israel, stands out with the highest co-citation count, significantly outpacing his peers. This preeminence is largely attributed to his groundbreaking research on the phenotypic polarization of TANs, published in 2009. His study, which contributed to the functional classification of TANs into N1 and N2 subtypes, has provided novel insights into the multifaceted role of TANs within the tumor microenvironment. This work has not only catalyzed further research in the field but has also become the most frequently cited literature, solidifying Fridlender’s status as a leading figure with the highest citation frequency, the most published articles, the highest H-index, and the strongest Total Link Strength among all authors. Furthermore, the research conducted by the GENTLES, A. J. team, as presented in ‘The prognostic landscape of genes and infiltrating immune cells across human cancers, ‘ published in 2015, is recognized for its second-highest citation frequency and the top average annual citation rate. Their large-scale data analysis has shed light on the significant interplay between gene expression and tumor-infiltrating immune cells in cancer prognosis. Subsequently, COFFELT, S. B. ‘s 2016 publication, ‘Neutrophils in cancer: neutral no more, ‘ which ranks third in both citation frequency and average annual citation, has also made a substantial impact on the understanding of the cancer immune microenvironment. In conclusion, scholars such as Jablonska, Jadwiga, and Fridlender, Zvi G., have exerted considerable influence in the TANs research domain. Fridlender’s pioneering work is particularly esteemed and widely acknowledged. Concurrently, the contributions of the GENTLES, A. J. and COFFELT, S. B. have been instrumental in advancing our knowledge of the cancer immune microenvironment.

As detailed in **Table 4** and illustrated in **Figure 6**, ‘Frontiers in Immunology’ emerges as a leading journal in the field, boasting both the highest publication count and a prominent position within the top 10 journals for co-citation frequency. ‘Cancer Research’ distinguishes itself with the highest co-citation frequency, securing the ninth rank in citation frequency overall. This distinction can be largely attributed to the substantial number of highly impactful articles featured in the journal. It is noteworthy that among the top 10 journals by co-citation frequency, there is a significant representation of specialized journals. Specifically, three are dedicated to oncology (‘Cancer Research’, ‘Cancer Cell’, ‘Clinical Cancer Research’), two to immunology (‘Journal of Immunology’, ‘Frontiers in Immunology’), and three are multidisciplinary scientific journals (‘Nature’, ‘Cell’, ‘Proceedings of the National Academy of Sciences, USA’ or ‘PNAS’). Additionally, ‘Nature Medicine’ and ‘Journal of Biological Chemistry’ are associated with biology and immunology, respectively, while ‘Nature’, ‘Cell’, and ‘PNAS’ are linked to molecular biology. This distribution aligns with the biplot analysis presented in **Supplementary Figure 1**, which underscores the interdisciplinary nature of the research landscape. The analysis of the journal heat map reveals the dynamics of heat within the field of scholarly publishing, from an initial tendency to publish in prestigious journals with high impact factors, to a later preference for high open access journals, and ultimately a renewed focus on those high impact journals. This fluctuation reflects the progression of research in TANs, with high-quality research generating attention and open journals helping to spread the field of research, further contributing to the emergence of higher-quality research.

The analysis of specific literatures reveals the key role of these literatures in the field of TANs research. As shown in **Table 6** and **Figure 9**, “Polarization of Tumor-Associated Neutrophil Phenotype by TGF-β: N1 versus N2 TANs” is undoubtedly the most authoritative article in this research area. It has the highest citation frequency and is closely linked to other highly cited literature, as well as being the first to explode with citations. “Tumor-associated neutrophils stimulate T cell responses in early-stage human lung cancer” and “ Hyperglycemia Impairs Neutrophil Mobilization Leading to Enhanced Metastatic Seeding” were the two articles with the strongest outbreaks and longer durations, suggesting that they also have high impact. “Neutrophil diversity and plasticity in tumor progression and therapy” is a review with a high citation rate that also cites many highly cited articles, which is a good source of information for readers to understand the field of TANs research.

### 4.2 Hotspots and frontiers

Keyword analysis is instrumental in discerning the frontiers and focal points within a field of study. In this research, a comprehensive keyword analysis was performed to delineate the predominant trends and temporal shifts in the domain of Tumor-Associated Neutrophils (TANs). The principal keywords identified include ‘neutrophil’, ‘tumor-associated neutrophils’, ‘tumor microenvironment’, ‘tumor’, ‘immunotherapy’, ‘NETs’ (Neutrophil Extracellular Traps), ‘metastasis’, ‘inflammation’, and ‘tumor-associated macrophages’ (as listed in **Table 5**). These keywords predominantly pertain to the biological functions of TANs, their roles within the tumor microenvironment, and their implications in immunotherapy and inflammatory responses, indicating that these topics are currently at the forefront of TANs research. The co-occurrence network diagram elucidates that high-frequency keywords such as ‘immunology’, ‘oncology’, and ‘inflammation’ have been central to past research endeavors. Within the immunology sphere, keywords like ‘immune cells’, ‘immune evasion’, ‘immune checkpoints’, ‘prognosis’, and ‘macrophage’ have been prominent. Similarly, in oncology, ‘tumor-associated neutrophils’, ‘metastasis’, ‘anti-tumor immunity’, and ‘tumor-associated macrophages’ have garnered significant attention. In the context of inflammation, ‘neutrophil’, ‘inflammation’, ‘innate immunity’, and ‘granulopoiesis’ have been frequently discussed. The recurring terms ‘neutrophil’ and ‘tumor-associated neutrophils’ underscore the pivotal role of neutrophils in the tumor microenvironment, as well as their potential contributions to tumor progression and immunomodulation. Furthermore, the recurrent mention of ‘tumor microenvironment’ and ‘immunotherapy’ underscores the significance of the immune context in cancer therapeutics. Notably, breast and colorectal cancers have emerged as the most active disease areas within TANs research, with their high frequency and Total Link Strength indicating a central position in neutrophil-related studies. This prominence may be attributed to the elevated incidence and mortality rates associated with these cancers, drawing considerable research focus. Employing VOSviewer software for visual mapping, the research on TANs was categorized into seven major directions. These clusters not only highlight the diversity of research but also suggest potential interconnections between different research avenues. For instance, the interplay between inflammatory responses and tumor development, along with the part played by immunomodulation in tumor immunity, are areas meriting further in-depth exploration in future studies. Keyword impact and heatmap analysis also shed light on the evolution of research trends and the shift in scientific interest. Keyword impact analysis illustrates that tumor-promoting research on TANs is a very hot topic. Heatmap analysis, on the other hand, reveals emerging research areas in TANs research (e. g., Nets, IMMUNOTHERAP, etc.), and those keywords appearing in multiple clusters, such as INNATE IMMUNITY, MYELOID-DERIVED SU, which not only show a consistently high level of interest in a specific area, but also connect different research topics. The heat of these keywords suggests that they may represent critical nodes in the disease process or potential targets for therapeutic intervention. For example, INNATE IMMUNITY relates to the first response of the intrinsic immune system, whereas MYELOID-DERIVED SU is associated with immunosuppression in the tumor microenvironment. TUMOR and TUMOR-ASSOCIATED M are directly linked to tumor progression and metastasis, whereas ANGIOGENESIS is a key process in tumor growth and spread.

### 4.3 Hot research and future prospects of TANs

The following aspects of current research on tumor-associated neutrophils (TANs) will be discussed: the origin of TANs, keyword hotspots in the field of TANs research, the anti-tumor and pro-tumor functions of TANs, and the pathogenic factors underlying the polarization and functional differences of TANs. Neutrophils, the most abundant polymorphonuclear leukocytes (56), are crucial for the innate immune system and have a significant role in tumor microenvironments (TME) (57). Originating from granulocyte-monocyte progenitors (GMPs) in the bone marrow (58), neutrophils mature and are mobilized into the bloodstream by Granulocyte colony-stimulating factor (G-CSF) (59). TANs result from neutrophil development and infiltration into the TME, primarily controlled by the chemokine receptor type (CXCR2) axis (60). In the TME, various cellular constituents release CXCR2 ligands, such as CXCL1-8, creating a chemotactic gradient that guides neutrophils to the tumor (61, 62). Kyle J. Eash et al. have shown that CXCR4 and CXCR2 are essential for controlling neutrophil release, with only fully differentiated neutrophils expressing CXCR2 gaining entry into the bloodstream and subsequently infiltrating target tissues (63, 64).Additional studies corroborate that SMAD4 deletion promotes the recruitment of TANs through the CXCR2 axis (65). SMAD4 deletion promotes colorectal cancer (CRC) expression of C-C Motif Chemokine Ligand 15 (CCL15) and recruits the CCR1 TAN (CCL15-CCR1 axis) with arginase-1 (ARG-1) and matrix metalloproteinase 9 (MMP-9) activities, thereby forming a pre-metastatic niche for disseminated tumor cells (e. g., in the lungs) (20, 66, 67). Interleukin-8 (IL-8), overexpressed in multiple cancer types, recruits neutrophils to the TME through CXCR1 and CXCR2, influencing TAN formation (68–70). Jing He et al. showed that IL-8 secretion, induced by METTL3 disruption, promotes TANs recruitment and regulates tumor growth (71). Yang’s research highlights IL-8’s role in TANs recruitment and JAG2 expression, and the blockade of CXCR2 signaling reduces tumor growth and TANs numbers while enhancing CD8+ T cell activity (72). Collectively, these studies underscore the pivotal role of the CXCR2-IL-8 axis in mediating the recruitment of TANs within the TME. Metastatic tumors, on the other hand, induce chemotaxis of circulating neutrophils by secreting large amounts of G-CSF; these recruited neutrophils are mostly immature and immunosuppressive, promoting cancer metastasis (73).Actually, only fully differentiated neutrophils expressing CXCR2 are permitted to enter the Circulatory system and subsequently infiltrate the corresponding tissues. G-CSF has been demonstrated to facilitate the proliferation of neutrophil precursor cells, resulting in the expansion and infiltration of immature neutrophils in the peripheral blood. This phenomenon does not contravene the complete differentiation of CXCR2. Myeloid-Derived Suppressor Cells (MDSCs) is a term used to describe a population of myeloid-derived non-lymphoid immunosuppressive cells that are enriched in cancer patients (74). G-MDSCs, which share surface markers such as CD11b with TANs, have been observed to differentiate into CD11b+/CD66b+ TANs in gastric cancer, a process linked to immunosuppression and tumor metastasis (75). It may suggest that when the body is in a state of urgency, G-MDSCs, originating from the expansion of immature myeloid cells (IMCs) in the bone marrow, migrate to peripheral tissues where they are transformed into TANs by cytokines like TGF-β present in the TME. Myeloid-related proteins (MRPs), specifically S100A8 and S100A9, are implicated in neutrophil migration, with high expression levels observed in the TME and pre-metastatic niche (51, 76). The spleen is also a significant source of TANs, which contribute to tumor progression by mobilizing immature myeloid cells that differentiate into tumor-associated macrophages (TAMs) and TANs (77, 78). These cells promote tumor growth and metastasis through cytokine secretion. Although the current study has not directly demonstrated the impact of splenic regulation of TANs on tumor therapy, it does highlight the critical role of the spleen in tumor-associated immune cell generation and demonstrates the importance of the spleen as a potential therapeutic target. The transition of neutrophils into N2 TANs is intimately linked to their role in facilitating tumor growth, irrespective of whether this occurs via the bone marrow-circulating-TME axis or the splenic route. Our keyword analysis from the existing literature highlights ‘chemokine’ and ‘immune evasion’ as prominent terms, underscoring the pivotal role of TANs in tumor immune evasion. Although the number of studies on the induction and development of N1 TANs is relatively limited, the phenomenon of immunosculpting or immunoediting – defined as the crosstalk between immune cells and tumor cells - indicates the potential for such interactions to alter tumor biological phenotypes. This concept suggests that the limited research on N1 TANs may be overlooking a crucial aspect of how these cells contribute to the dynamic immune-tumor interface (79). It has been demonstrated that tumor cells can influence the secretion of molecules by neutrophils, which in turn promote tumor growth. To illustrate, breast cancer cells secrete GM-CSF, which induces neutrophils to produce oncostatin M, a protein that boosts VEGF production and cancer cell invasion (80). However, under effective immunotherapy, there is an increase in the number of neutrophils present in tumors, accompanied by the expression of interferon-stimulated genes (ISGs), which exhibit antitumor functions. The transcription factor IRF1 in neutrophils is pivotal for an efficacious antitumor response; its absence precludes the efficacy of immunotherapy (81). Furthermore, a study identified a crosstalk between tumor-associated neutrophils (TANs) and CRC cells through the AGR2-CD98hc-xCT axis, which enhances CRC cell migration and creates a feedback loop driving metastasis (7). These results point to the potential of TANs in cancer therapy, suggesting that they can be mobilized against cancer cells rather than simply promoting tumor growth. Therefore, strategies to modulate TANs may be a new way to improve the efficacy of cancer immunotherapy.

As illustrated in **Figure 7**, the relationship between TANs and “Cancer” is one of the most rapidly evolving areas of research in recent years. As a critical component of the tumor microenvironment, TANs exert a profound influence on tumor progression and metastasis (82–86). TANs have been demonstrated to exert a regulatory influence on tumor growth, with their presence observed in a wide range of solid tumors, including metastatic melanoma (87), bronchoalveolar carcinoma (88), renal carcinoma (89), head and neck squamous cell carcinoma (HNSCC) (90), pancreatic cancer (16), and gastric cancer (91), has been identified as a marker of a poor prognosis in a number of clinical and laboratory studies. In the context of these specific types of cancer, neutrophils have been observed to exhibit tumor-promoting properties that may potentially be harmful to the host. Among these, hepatocellular carcinoma, pancreatic cancer, and gastric cancer have emerged as key diseases in our identified hot research areas. The term ‘TME’ has been referenced 85 times in the keywords over the past fifteen years, which serves to illustrate its significance. TME exerts control over neutrophil recruitment through the operation of specific molecular mechanisms. On the other hand, the accumulation of TANs in TME is intimately linked to tumor invasiveness and metastatic progression. Song et al. revealed that in hepatocellular carcinoma(HCC)-TME, cancer associated fibroblasts (CAF) -derived cardiotrophin-like cytokine factor 1 (CLCF1) increased the paracrine secretion of CXCL6 and TGF-β in tumor cells, thereby promoting the infiltration and polarization of TANs (92). In clinical samples, upregulation of the CLCF1-CXCL6/TGF-*β* axis was strongly associated with the emergence of cancer stem cells, increased “N2”-polarised TANs, high tumor stage and poor prognosis (92). A transcriptional study in NSCLC has identified a TANs cluster characterized by overexpression of high mobility group box 1 (HMGB1). This cluster is hypothesized to interact with the TME via HMGB1-TIM-3 interactions, potentially suppressing antitumor immunity and facilitating immune evasion through the GATA2/HMGB1/TIM-3 signaling axis (93). These findings collectively indicate that TANs and TME exhibit multifaceted roles in relation to one another and that they may potentially share common pathologic mechanisms across diverse cancer types. The ratio of tumor-infiltrating neutrophils to lymphocytes (NLR) is a valuable indicator for assessing the prognosis of cancer patients, reflecting the immune status in the TME, which is also one of the hottest keywords we have found. A higher neutrophil-to-lymphocyte ratio (NLR) is associated with a poorer prognosis in numerous types of cancer (52, 94–98). In a study of uroepithelial carcinoma of the bladder, the presence of neutrophils and NLR were associated with high-grade uroepithelial tumors, TANs were associated with tumor grade and stage, and TALs (especially CD8 T cells) and NLR were more likely to be associated with progression of tumor invasion in this study (99). Chen et al. demonstrated that a low N1/N2 ratio was associated with poorer tumor differentiation, easier lymph node metastasis, and a higher TNM stage (100). Conversely, a high N1/N2 ratio was identified as an important prognostic indicator for overall survival (OS) and recurrence-free survival (RFS). Additionally, tumor-associated N1/N2 neutrophils exhibited an inverse correlation with tumor-infiltrating CD8+ T cells and Tregs. In conclusion, the inverse correlation between TANs and lymphocytes may facilitate a deeper understanding of the immune system and its functioning, and thus merits further investigation.

The anti-tumor and pro-tumor mechanisms of TANs have now been more widely demonstrated. Firstly, it is established that tumor-associated neutrophils (TANs) can directly kill tumor cells through self-exposure and cytotoxic effects (101). It is noteworthy that this killing effect is associated with local hypoxia but not with T cells, TANs recruitment is reduced after hypoxia is relieved and TANs under these conditions is more capable of killing tumor cells (102). TANs also have indirect anti-tumor effects, as they can stimulate adaptive anti-tumor immune responses by promoting the recruitment of other immune cells and having antigen-presenting potential themselves (102, 103). Vono et al. demonstrated that neutrophils isolated from vaccine-draining lymph nodes of rhesus monkeys exhibited HLA-DR expression and were capable of presenting vaccine antigens to autologous antigen-specific memory CD4+ T cells *in vitro* (104). This suggests that neutrophils may function as antigen-presenting cells (APCs), leveraging their abundance in the immune system to potentially regulate antigen-specific T cell responses. Neutrophils can recruit and activate T cells by secreting cytokines such as TNFα, and histone G promotes T cell proliferation and cytokine production (69, 105). For instance, TANs secrete human mast cell chymotrypsin (HC) and human neutrophil histone G (hCG), both of which readily cleave two interleukin-1 (IL-1)-associated alerting proteins, interleukin-18 (IL-18) and interleukin-33 (IL-33), as well as the cytokine interleukin-15 (IL-15), which is important for T-cell homeostasis (106). TANs also kill tumor cells by generating reactive oxygen species (ROS), with hypochlorous acid (HOCl) playing a major role in recognizing the surface of target cells and mediating tumor cell lysis by a mechanism dependent on leukocyte function-associated antigen 1 (LFA-1) (107). In addition, the distinctive adhesion pathway mediated by the upregulation of CD11b/CD18 on activated neutrophils allows these cells to adhere to the vascular endothelium and form a sub-neighborhood microenvironment, which allows for the local aggregation of oxidants and proteolytic enzymes in concentrations sufficient to cause endothelial damage and matrix degradation (108, 109). Another interesting study have shown that neutrophil-produced H2O2 activates transient receptor potential cation channels (TRPM2), resulting in the uptake of lethal levels of calcium ions by tumor cells (110). Furthermore, TRPM2 expression is up-regulated in cancerous tissues, making these cells more susceptible to the cytotoxic effects of neutrophils (110). In addition to ROS toxicity, TANs also induce tumor cell death by promoting the expression of nitric oxide synthase (iNOS) and the release of nitric oxide (NO) via hepatocyte growth factor (HGF) (111). Notably, superoxide itself is not directly involved in cell killing; instead, catalase (which converts H2O2 to H2O and O2) completely inhibits cell killing (11). In addition, TANs can directly kill tumor cells via antibody-dependent cytotoxicity (ADCC), which is achieved by neutrophils through the expression of Fc receptors that mediate ADCC and may mechanically disrupt tumor cell membranes through interactions with signal-regulated protein *α* (SIRP*α*) and CD47 (54, 55). This phenomenon has been found in a variety of cancers (including non-Hodgkin’s lymphoma, breast cancer and B-cell lymphoma) (112–114). In a mouse model of cervical adenocarcinoma, TANs secrete proteases that induce tumor cell detachment from the basement membrane, thereby inhibiting tumor growth and metastasis. Despite the evidence from these studies indicating that TANs have an anti-tumor function, neutrophils are primarily known to have an immunosuppressive effect (55). Once TANs are activated within the tumor microenvironment, they significantly enhance the inflammatory environment and drive tumor progression through a series of complex mechanisms. The release of large quantities of interleukin-8 (IL-8) by inflammatory cells has two main effects. Firstly, it promotes the survival of TANs, and secondly, it attracts more neutrophils to accumulate at the tumor site, thus exacerbating the inflammatory response (115). The upregulation of IL-8 and neutrophil enrichment in KRAS-mutant CRC tissues has been demonstrated, which suggesting that exosomes may transfer mutant KRAS to recipient cells and trigger increases in IL-8 production, neutrophil recruitment and formation of NETs, eventually leading to the deterioration of CRC (50). In contrast to the anti-tumor function of ROS described above, TANs are able to increase tissue sensitivity to carcinogens by releasing ROS and RNS and mediating genotoxicity. The research conducted by Stefanie K. Wculek and colleagues indicates that neutrophils amplify the genotoxicity of ethyl carbamate in lung cells through the generation of ROS, and this process directly facilitates tumor transformation, with ROS-dependent DNA damage being temporally confined to ethyl carbamate exposure and distinctly unrelated to extensive tissue damage or inflammation (116). In 2019, Veronika Butin-Israeli identified a novel mechanism of genotoxicity that, interestingly, does not rely on ROS. In contrast, TANs facilitate the formation of double-strand breaks (DSBs) in epithelial DNA through the release of pro-inflammatory microRNA particles (miR-23a and miR-155), and the accumulation of DSBs in injured epithelial cells subsequently results in genomic instability, impaired tissue healing, and the promotion of tumorigenesis (117). Prostaglandin E2 (PGE2) or neutrophil elastase (NE) can directly promote the proliferation of tumor cells. A. McGarry Houghton have demonstrated that NE induces degradation of insulin receptor substrate-1 (IRS-1) in tumor cell endosomes, as NE degraded IRS-1, there was increased interaction between phosphatidylinositol 3-kinase (PI3K) and the potent mitogen platelet-derived growth factor receptor (PDGFR), thereby skewing the PI3K axis toward tumor cell proliferation (118). The release of MMP-9 is associated with the promotion of tumor angiogenesis and plays an important role in extracellular matrix(ECM) remodeling and membrane protein cleavage (119). A study of prostate cancer has revealed the molecular mechanism by which MMP-9 regulates tumor cell invasion and metastasis. It has been indicated that MMP-9 enhances prostate cancer cell invasion by specifically degrading serpin protease nexin-1 (PN-1) and deregulating the inhibitory effect of PN-1 on urokinase plasminogen activator (uPA) (120). Whereas in the study by Lukas et al. neutrophil-derived MMP-9 was found to mediate the release of larger VEGF isoforms not through cleavage but rather, and they demonstrated that MMP-9 was able to release biologically active VEGF165 from the ECM of colon cancer cells via cleavage of acetylheparin sulfate, which promotes tumor angiogenesis (121). In addition, the immunosuppressive capacity of neutrophil subpopulations has all been associated with tumorigenesis (122). In conclusion, it can be stated that TANs play an important role in several key aspects of tumor malignant transformation, progression, extracellular matrix remodeling, angiogenesis, cell migration and immunosuppression. This is achieved by degrading the extracellular matrix, inhibiting immune responses, stimulating tumor cell proliferation, increasing tumor metastatic potential and promoting angiogenesis, which in turn promotes tumor progression.

The dual effect of TANs can also be observed in another keyword: Neutrophil Extracellular Trap Networks(NETs). On the one hand, TANs are involved in anti-tumor immune responses by releasing NETs. NETs are capable of capturing and confining tumor cells, while they contain antimicrobial proteins and enzymes (e.g. myeloperoxidase MPO and neutrophil elastase NE) that directly kill tumor cells (86, 123, 124). Moreover, NETs facilitate tumor immune surveillance by stimulating dendritic cells and augmenting T cell-mediated immune responses (125, 126). Conversely, NETs may also be involved in tumor progression by promoting tumor cell invasion and migration. The reticular structure of NETs may provide a physical adhesion platform for circulating tumor cells (CTCs), thereby promoting tumor cell colonization and metastasis in distal organs (127). Moreover, the enzymes present within NETs are capable of degrading the extracellular matrix, thereby facilitating the spread of tumor cells (128, 129). It is noteworthy that the oncogenic role of NE has been demonstrated in lung, prostate, and colon cancer (118, 130–133). With respect to tumor immune evasion, NETs may facilitate tumor cell evasion of immune surveillance by forming a physical barrier that impedes immune cell recognition and attack. Additionally, NET discharge may modify chemical signals within the tumor microenvironment, influencing immune cell polarization and functionality, and consequently, the equilibrium of the tumor immune response (134).

As previously discussed, the contrasting roles of N2 TANs in promoting tumor formation and N1 TANs in exerting antitumor effects have been delineated with reasonable clarity. However, the underlying factors that mediate these dichotomous effects of TANs remain unclear. Consequently, investigating these factors will constitute a pivotal research focus in future studies of TANs. The hypothesis that TANs are classified as N1/N2 types has been corroborated by further research on TANs. The study by Mareike Ohms et al. was successful in polarizing human neutrophils into N1/N2 types *in vitro*, and it could show functional and phenotypical differences between neutrophils cultured in the presence of N1- or N2-polarizing cocktails (10). In present study, scientists have identified a number of molecules that can be used to differentiate between N1 and N2. The N1 markers include intercellular cell adhesion molecule-1 (ICAM-1), inducible nitric oxide synthase (iNOS), C-C motif ligand 3 (CCL3), and TNF-α, among others. The N2 markers include CCL17, CCL2, Arg, CCL5, and vascular endothelial growth factor (VEGF) (135, 136).The role of transforming growth factor-β (TGF-β) signaling within the tumor microenvironment (TME) has been implicated in the promotion of a pro-tumorigenic neutrophil phenotype (N2) (137). In contrast, type I interferon (IFN) signaling or the blockade of TGF-β signaling has been shown to direct neutrophils toward an antitumor phenotype (N1) (138).The significance of these two pivotal inducing factors is further underscored by the data presented in **Figures 7A, B**. Moreover, ongoing research continues to uncover additional factors that modulate the polarization and functional profile of TANs. For instance, Chung et al. revealed that Smad3 activation in TANs is associated with the predominant N2 polarization status and poor prognosis of non-small cell lung carcinoma (NSCLC) patients, while they proposed CD16b/iNOS and CD16b/CD206 as markers to identify human N1 and N2 TANs (139). This discoveris may resolve the inability to distinguish the two subtypes from surface markers, but further experiments are required to validate this conclusion. Luo et al.’s study disclosed that the expression of N2-specific marker genes was significantly reduced in TANs following pretreatment with 4-phenylbutyric acid. This observation suggests that the pro-tumorigenic capabilities of TANs may be diminished when endoplasmic reticulum stress is not activated. Therefore, it is plausible to posit that the activation of the endoplasmic reticulum could be implicated in the phenotypic shift of TANs toward the N2 state (140).Wang et al. showed that HCC cell-derived CXCL9 promotes N1 polarization of neutrophils *in vitro*, while the specific CXCR3 inhibitor AMG487 significantly blocked this process (141).These findings provide further evidence for the dual effects of TANs and suggest that TANs may directly or indirectly affect patient survival and prognosis. It would be beneficial for future studies to consider comprehensive analyses covering multiple cancer types in order to explore the heterogeneity of the phenotypic distribution of TANs, which would help to deepen our understanding of the functional and clinical relevance of TANs. Although both typologies of TANs are now generally recognized, recent studies suggest that a simple dichotomy of immune cells in cancer may not provide a comprehensive description of TANs. A study utilizing time-of-flight mass spectrometry (CyTOF) analysis of cytometry has demonstrated the existence of at least seven subpopulations of mature neutrophils that differ in surface markers and function in individuals with cancer (142). It can be posited that a variety of different anti-tumor and pro-tumor effects may be exhibited by different phenotypes of mature neutrophils in the context of TANs. A unique subset of HLA-DR TAN with anti-tumor capacity is also detected in early stages of human lung cance, the subpopulation, exhibiting characteristics of both granulocytes and antigen-presenting cells like dendritic cells and macrophages and termed ‘Hybrid TANs, ‘ is capable of effectively inducing T-cell responses, encompassing both tumor antigen-specific and non-specific immunity (115, 143). Notably, the number of such hybrid TANs was found to be decreased in large tumors, which appeared to be due to an associated hypoxic TME (143). Given the considerable heterogeneity and plasticity of TANs within the TME, accurate subpopulation analysis of TANs has become an important research focus (55). However, it is important to note that neutrophils in cancer are not limited to TANs but also include numerous subpopulations in the bone marrow and circulation (144). To date, the extensive heterogeneity of neutrophils in cancer remains a topic worthy of further study.

The interaction of TANs with a variety of other cell types in the tumor microenvironment, including TAMs, platelets, natural killer (NK) cells and T cells, forms a complex network that influences tumor development and metastasis. The terms “TAMs” and “T cell” are both significant keywords that are highlighted on the keyword hotspot map. Although there is no direct evidence that TANs and TAM interact via MPO and MMR, there is already evidence that a large MPO-positive neutrophil infiltrate is found in colorectal (145) and lung cancers (146), with high levels of macrophage mannose receptor (MMR) expression by M2-like macrophages (42). Whereas MPO binding to MMR induces secretion of reactive oxygen intermediates, IL-8, TNF-gr and GM-CSF in chronic inflammatory environments (e. g. rheumatoid joints) (147). This may suggest that TANs and TAMs co-exist in a specific way in the tumor microenvironment, promoting an inflammatory response in the tumor microenvironment. A study revealed a correlation between elevated NLR and elevated CCL2 expression in tumor tissues (53). Additionally, the conditioned medium of TANs and recombinant CCL2 and CCL17 were observed to enhance the migration of macrophages derived from HCC patients or mice. These findings collectively indicate that TANs and TAMs interact through chemokines, such as CCL2, and collectively promote tumor growth and metastasis. Interestingly, the recruitment of TAMs by TANs to the appropriate regions in turn regulates the function of TANs, similar to the interaction between neutrophils and macrophages in the inflammatory environment. In addition to direct inhibition of T cells via ROS, iNOS and mediators such as ARG1, TANs can also inhibit T cell anti-tumor immunity by recruiting TAMs and regulatory T cells (Tregs) to remodeling of the TME via CCL17 and CCL2 (53). TANs inhibit T cells by expressing programmed cell death-ligand 1 (PD-L1) to suppress anti-tumor response. In contrast, blockade of PD-1/PD-L1 resulted in reduced immunosuppression of T cells and enhanced infiltration and activation. Zhang et al. found that after tumorigenesis, TANs displayed N2-like state and secreted cytokine IL-10 to promote the activation of c-Met/STAT3 signaling, while the transcription factor STAT3 increased the level of PD-L1 in tumor cells and promoted the polarization of neutrophils toward N2-like state. granulocyte polarization toward an N2-like state, leading to a positive feedback loop between TANs, IL-10, STAT3, PD-L1, and TANs themselves (148). Inhibiting one of the processes in the positive feedback pathway may prove beneficial in the treatment and prognosis of the tumor. Michaeli et al. reported that TANs promote immunosuppression by strongly inducing CD8+ T cell apoptosis, which leads to tumor progression, and that the TANs-induced CD8+ T cell death mechanism involving the TNF signaling pathway and NO production (149). In contrast, it has been reported that TANs can promote CD8+ T-cell recruitment and activation by producing T-cell chemoattractant (e. g., CCL3, CXCL9, and CXCL10) and proinflammatory cytokines (IL-12, TNF-α, and GM-CSF) (69). The question of how to regulate the production of T cell-promoting factors by TANs and reduce the production of suppressors is also a topic of discussion. In the early stages of lung cancer, crosstalk between TANs and activated T cells resulted in significant upregulation of CD54, CD86, OX40L, and 4-1BBL co-stimulatory molecules on the surface of neutrophils, which promoted T cell proliferation in a positive feedback loop. This result suggests that the upregulation of co-stimulatory molecules on TANs enhances T cell immunity, whereas the upregulation of PD-L1 suppresses T cell responses (115). Modulating specific signaling molecules in the microenvironment to direct TANs toward a phenotype that promotes T cell immunity, or monitoring changes in surface molecules of TANs during tumor treatment, could serve as valuable strategies for assessing therapeutic efficacy and predicting alterations in the tumor’s immune response.

Platelets are the first site to appear in the inflammatory process that accompanies the development of cancer. No influx of monocytes, lymphocytes, dendritic cells or NK cells was observed in the early stages of metastasis formation (150). It appears that neutrophil recruitment to the tumor microenvironment is dependent on platelet activation. This process does not occur when the function of these cells is impaired or when platelets are reduced. The function of platelets in the formation of TANs can be considered in two distinct ways. Firstly, platelets release the chemokine CXCL5/7, which binds to CXCR2 on the surface of neutrophils, thereby activating and migrating these cells (49). Secondly, platelets serve as a source of TGF-β, which plays a pivotal role in the development of N2 TANs (151, 152). Recent studies have also revealed a potential inhibitory effect of TANs and neutrophils on natural killer (NK) cell function during tumor development. The study by Sun et al. showed that TANs inhibit the cytotoxicity and infiltration capacity of NK cells through the PD-L1/PD-1 axis and regulate the expression of PD-L1 and PD-1 through the GCSF/STAT3 and IL-18 pathways, revealing the effect of neutrophils on NK cell dysfunction in the loaded state and its molecular mechanisms (153). Yang et al. revealed that tumor-associated neutrophils were able to influence macrophages, NK cells and T cells through IL16, IFN-II and SPP1 signaling pathways (154). The main mechanism may be the release of nitric oxide (NO) and ROS and arginase 1 (ARG1) activity by TANs to inhibit NK cytotoxicity and T cell proliferation (155, 156). The elucidation of these mechanisms provides new insights into understanding the complexity of immunosuppression in the tumor microenvironment, and future studies will need to further explore how intervention in these pathways can enhance the anti-tumor activity of NK cells or help NK cells escape suppression. TANs are actively involved in the recruitment of B cells to the TME in addition to their ability to produce extensive crosstalk with the aforementioned cells. Merav E. Shaul et al. have clarified that TNFα is the main cytokine in TANs-mediated B cell chemotaxis, that recruitment of CD45+B220+CD138- splenic B cells by TANs *in vitro* leads to B cell phenotypic modulation, and that *in vitro* experiments have confirmed the ability of TANs to induce B cell differentiation into IgG-producing plasma cells, and that the process is dependent on the surface of TANs. process is dependent on a B-cell activating factor (BAFF) contact mechanism on the surface of TANs (157). Interestingly we will find that TNFα tends to associate with N1-type TANs, which may suggest a novel immunoregulatory network between TNFα, N1-type TANs and B-cells, in which the interaction between TANs and B-cells is critical for the formation of tumor immune response. This interaction may, together with other immune cell types, such as T cells and dendritic cells, constitute a complex network of immune responses that collectively influence tumor progression and patient response to therapy.

Increasing evidence suggests that neutrophils play an active role in promoting tumor development. However, clinical applications are still limited to the systemic treatment of TANs to avoid neutropenia (158). The blockade of immune checkpoints of the neutrophil programmed cell death 1 (PD1)/PD-L1 pathway, targeted binding of CXCR2, CXCR4, G-CSF, TGF-*β*, etc., which in turn inhibits the recruitment, expansion and polarization of tumor neutrophils, may provide some ideas for neutrophil-targeted tumor therapies. In view of the above discussions, we propose a series of prospective research directions for the investigation of TANs (1): Elucidating the mechanisms that induce the polarization of TANs from the N2-type to the N1-type during their chemotactic migration (2). Investigating the shared pathological mechanisms between TANs and the TME across a spectrum of cancers (3). Determining whether there are variations in TAN subtypes observed among patients with different cancers (4). Identifying additional markers to differentiate between TAN subtypes, addressing the complexity and heterogeneity of TANs (5). Clarifying the intricate mechanisms of TAN interactions with other tumor-associated cells, such as TAMs, tumor-associated platelets, and T cells. These research avenues may provide insights into the role of TANs in tumorigenesis and inform the development of novel therapeutic strategies. We anticipate that subsequent research will leverage the complete antitumor potential of TANs and integrate existing effective antineoplastic therapies with targeted neutrophil interventions, thus offering a promising direction that could result in safer and more efficacious treatment strategies.

### 4.4 Limitations

This study is the first to use bibliometric visualization to analyze studies related to TANs over the past 20 years. However, this study inevitably has some limitations. First, the data used in this study were only from the WOSCC database, excluding data from other databases such as PubMed, Cochrane Library, and Google Scholar. Despite the comprehensiveness and reliability of WOSCC, there may be some missing literature in the data from the WOSCC database; only English language literature was included in this study, which may lead to biased results. There was also the inclusion of literature up to March 21, 2024, and subsequent publications were not included in the study in time. Secondly, the data in this study may be inconsistent in many ways, for example, the same institution may have used different names at different times; and the same author published papers in the field at different institutions. Finally, although this study provides a comprehensive overview of the research field of TANs, there are some limitations in the study of keywords. The keyword analysis relied primarily on the titles and abstracts of the literature, which may not have fully captured the depth of information in the full text of the articles; the setting of the minimum number of citations may have excluded some emerging but important research directions. Future research could further explore these limitations and utilize more comprehensive data analysis methods to provide deeper insights.

## 5 Conclusions

In this study, we used bibliometrics and visual analytics to conduct a comprehensive review and analysis of research on tumor-associated neutrophils (TANs) between 2000 and 2024. A comprehensive review was conducted through the Web of Science Core Collection (WOSCC) database, we systematically sorted out the global trends in TANs research, identifying key publications, core authors, and research institutions, as well as research hotspots and frontiers in the field. Frontiers in Immunology and CANCERS are influential journals in the field, and Fridlender, Zvi G. is a leading author in the field. The fields of immunology, oncology and inflammation are currently experiencing a surge in interest, with the extensive heterogeneity of TANs, the pro-tumorigenic function of the N2 type and its relationship with TME, various cancers, or crosstalk with other immune cells emerging as popular avenues for future research. This study elucidates the basic scientific knowledge of TANs and the relationship with tumors and other immune cells, also provides important clues to research trends and hotspots. We hope that this study will promote academic exchanges in the field of TANs research and help researchers better grasp the current general trends in the field.

## Data Availability

The original contributions presented in the study are included in the article/**Supplementary Material**. Further inquiries can be directed to the corresponding author.
