# Supplementary figures and images for: Global trends in tumor-associated neutrophil research: a bibliometric and visual analysis
Source: Front Immunol. 2025 Mar 14;16:1478092. doi: 10.3389/fimmu.2025.1478092 (PMC11949894; doi:10.3389/fimmu.2025.1478092)

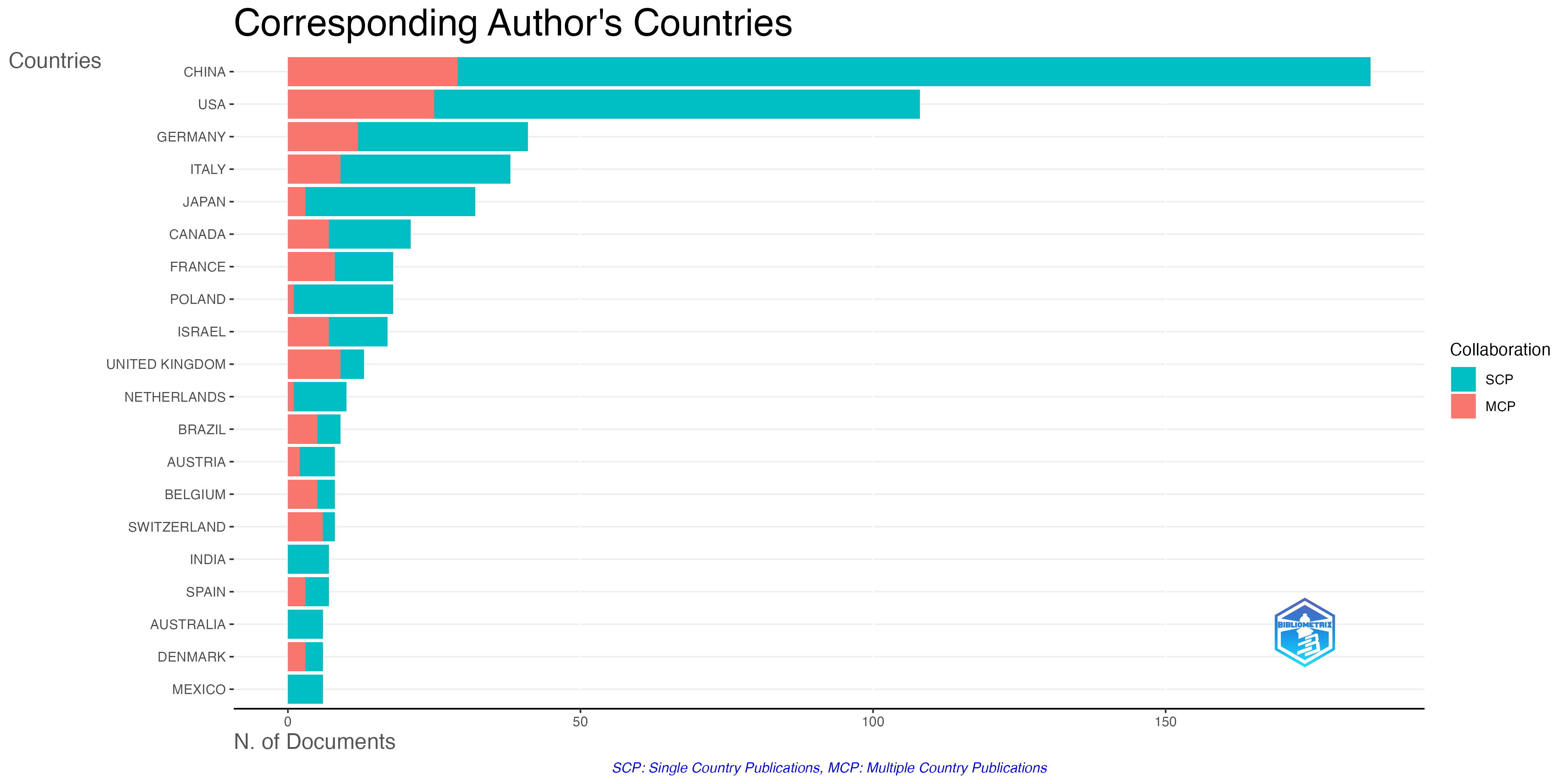

Supplement: Supplementary file 1 [file DataSheet1.zip › Supplemental Figure20250203/Supplemental Figure 1.png]

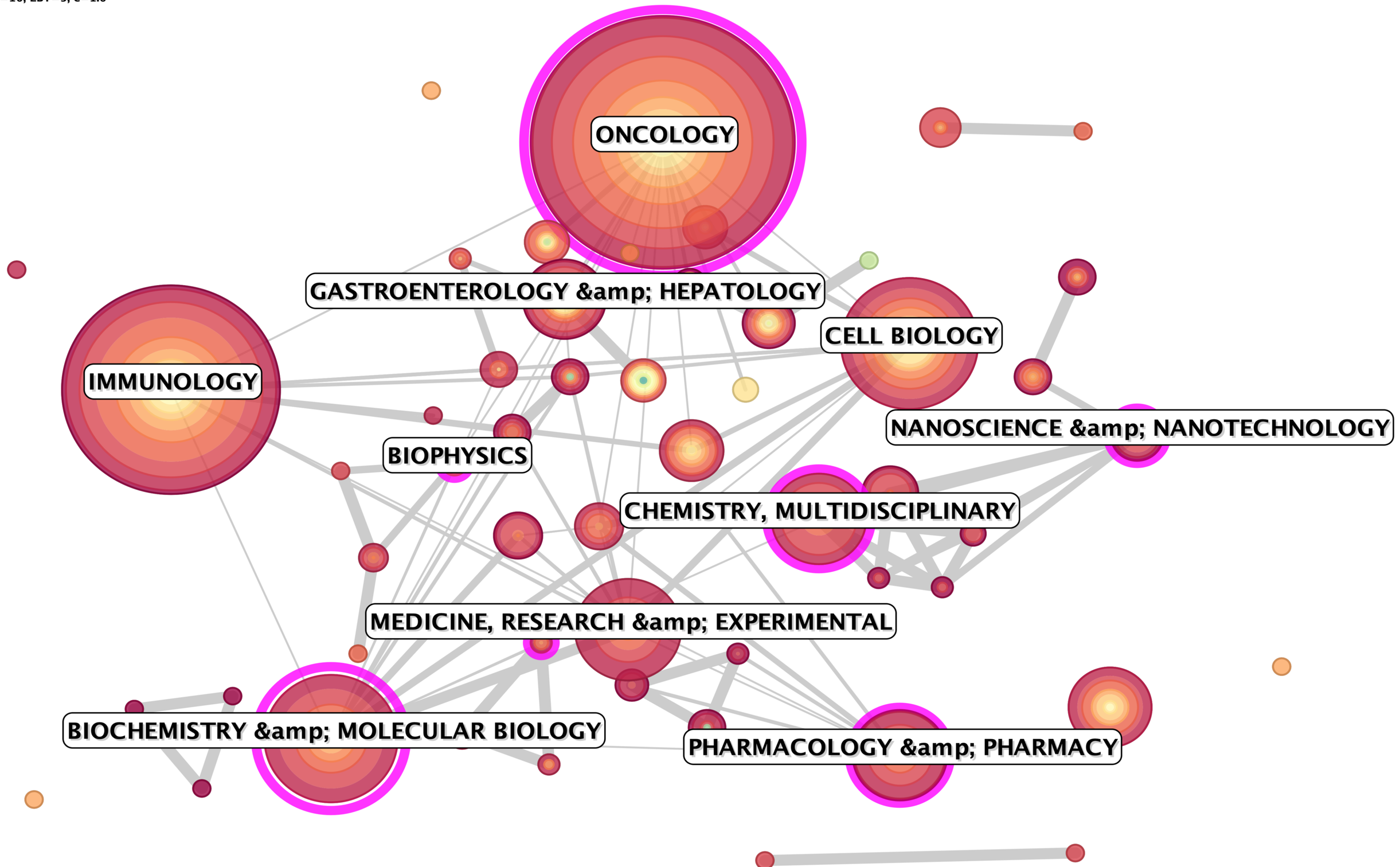

Supplement: Supplementary file 1 [file DataSheet1.zip › Supplemental Figure20250203/Supplemental Figure 2.pdf]
